# Exploring the Hospital Microbiome by High-Resolution 16S rRNA Profiling

**DOI:** 10.3390/ijms20123099

**Published:** 2019-06-25

**Authors:** Pabulo H. Rampelotto, Aline F.R. Sereia, Luiz Felipe V. de Oliveira, Rogério Margis

**Affiliations:** 1 PPGBCM, Center of Biotechnology, Federal University of Rio Grande do Sul, 9500, Porto Alegre, RS 91501-970, Brazil; prampelotto@hcpa.edu.br; 2 Laboratory of Experimental Hepatology and Gastroenterology, Hospital de Clínicas de Porto Alegre (HCPA), 2350, Porto Alegre, RS 90035-903, Brazil; 3 Neoprospecta Microbiome Technologies, 1302, Florianópolis, SC 88057-260, Brazil; aline@neoprospecta.com (A.F.R.S.); felipe@neoprospecta.com (L.F.d.V.O.)

**Keywords:** microbiota, nosocomial pathogens, hospital-acquired infections, 16S rRNA, clinical microbiology, Acinetobacter, Staphylococcus, Pseudomonas

## Abstract

The aim of this work was to analyze and compare the bacterial communities of 663 samples from a Brazilian hospital by using high-throughput sequencing of the 16S rRNA gene. To increase taxonomic profiling and specificity of 16S-based identification, a strict sequence quality filtering process was applied for the accurate identification of clinically relevant bacterial taxa. Our results indicate that the hospital environment is predominantly inhabited by closely related species. A massive dominance of a few taxa in all taxonomic levels down to the genera was observed, where the ten most abundant genera in each facility represented 64.4% of all observed taxa, with a major predominance of *Acinetobacter* and *Pseudomonas*. The presence of several nosocomial pathogens was revealed. Co-occurrence analysis indicated that the present hospital microbial network had low connectedness, forming a clustered topology, but not structured among groups of nodes (i.e., modules). Furthermore, we were able to detect ecologically relevant relationships between specific microbial taxa, in particular, potential competition between pathogens and non-pathogens. Overall, these results provide new insight into different aspects of a hospital microbiome and indicate that 16S rRNA sequencing may serve as a robust one-step tool for microbiological identification and characterization of a wide range of clinically relevant bacterial taxa in hospital settings with a high resolution.

## 1. Introduction

Hospital-acquired infections (HAIs) represent a serious public health problem, affecting millions of people worldwide [1]. Also known as nosocomial infections, they are the fifth leading cause of death in acute-care hospitals. In the United States, these infections cost several billions of dollars and result in approximately 90,000 deaths annually [2,3]. In developing countries, where the burden of endemic healthcare-associated infection is significantly higher [4], the prevalence of HAIs varies between 5.7% and 19.1% [1].

One of the challenges in preventing HAIs is understanding the microbial diversity associated with the hospital environment, the sources of infectious agents and the routes of transmission. Recent studies have suggested that environmental contamination plays a significant role in HAIs and several pathogens can persist for months in surfaces and serve as vehicles of transmission and dissemination in hospital facilities [5,6,7]. Thus, understanding the hospital microbiome could be essential to maintaining low levels of HAI infections and to help improving healthcare assistance.

DNA sequencing of the 16S rRNA gene has been successfully used for the characterization of microbial populations in a variety of habitats [8,9,10,11]. The advantage of this approach is that all microbial taxa may be detected and the limitations of culture conditions are easier to overcome [12]. Consequently, the application of such molecular methods has the potential to revolutionize the landscape of clinical microbiology and infectious diseases [13,14,15], and reveal which bacteria are present in hospitals and how they interact with each other and the environment. For these reasons, metagenomic studies involving both whole-genome sequencing and targeted gene sequencing are necessary to characterize in detail the microbial communities associated with hospital environments.

With the advent of next generation sequencing technologies, which have allowed the massive parallel sequencing of the 16S rRNA gene, the identification and tracking of bacterial diversity in hospital environments has become feasible. These pioneer studies have demonstrated the potential use of amplicon sequencing to identify a variety of pathogens associated with the development of HAIs [16,17,18,19]. Nevertheless, the information obtained from them is limited and sparse, i.e., usually based on few samples when considering the complex environments of a hospital. A comprehensive view of the hospital microbiome will help us better understand different aspects concerning the microbial ecology of this heterogeneous environment, which is subject to different selective pressures and still poorly explored. Furthermore, it will be essential for the development of the new practices and preventive measures needed to significantly reduce the high rates of hospital infections.

In order to identify new patterns that help better characterize the hospital microbiome, the aim of this work was to analyze and compare the bacterial communities from different inanimate surface environments of a Brazilian teaching hospital using high-throughput sequencing of the 16S rRNA gene. Overall, our results indicated that the hospital microbiome presents a homogeneous structure composed of a massive dominance of a few taxa in all taxonomic levels and a microbial network with low connectedness forming a clustered topology.

## 2. Results

### 2.1. Library Characterization

For bacterial community profiling at high-resolution and with high accuracy, we applied a rigorous filtering process at different depths and levels before any taxonomic analysis (in particular, the removal of operational taxonomy units (OTUs) with less than 5 reads during the clustering process and the removal of spurious OTUs later). The resulting library, after all filtering steps, is summarized in Table S1.

In total, 663 samples were collected during the six months. 502 (75.7%) samples contained classifiable sequences, while 161 (24.3%) did not present sequences after the filtering process. It is worth nothing that the library presented some particular features that were taken into account before further analysis. First, a relatively low number of sequences in most libraries was observed, which can be partially related to the rigorous filtering process, but most importantly, due to the intrinsic feature of the hospital environment presenting only a few number of microorganisms (when compared to other environments), as a consequence of its constant cleaning and sterilization procedures. Second, a large variability in library sizes across samples was observed, which indicated that rarefying the libraries with a non-parametric test should be the chosen method of normalization.

### 2.2. Composition of Bacterial Communities

After all trimming steps, the resulting library composed of the 502 samples contained 7,925,186 classifiable sequences grouped in 878 OTUs, which belong to 567 species and 203 genera. This dataset of high quality classifiable sequences was used to compute the final OTU table. Due to such high variability in library size, the raw OTU table was normalized using CSS.

**Figure 1:**
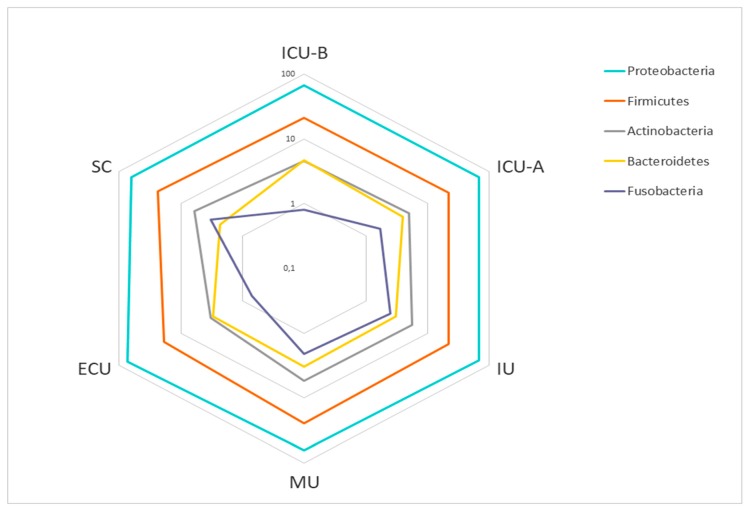
Abundance of the main phyla in the hospital facilities. Relative abundances are shown as percentages (%) on a log scale. Abbreviations: ECU, Emergency Care Unit; MU, Medical Unit; SC, Surgery Center; IU, Inpatient Unit; ICU-A, Intensive Care Unit A; ICU-B, Intensive Care Unit B.

The analysis of the sequences showed the presence of only five phyla (Figure 1). A major dominance of Proteobacteria was observed in all facilities (67.5%), with smaller proportions of Firmicutes (22.0%), Actinobacteria (5.0%), Bacteroidetes (3.4%), and Fusobacteria (1.9%). Such dominance of a few taxa was observed in all taxonomic levels. The ten most abundant genera in each facility represented 64.4% of all observed taxa (Table 1), with major predominance of *Acinetobacter* and *Pseudomonas*.

### 2.3. Structure and Diversity of Bacterial Communities

**Figure 2:**
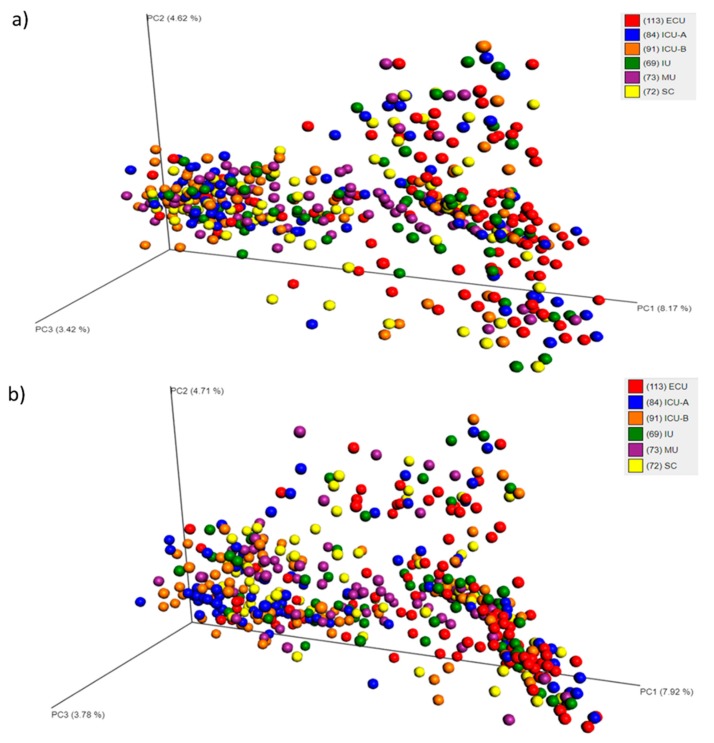
Principal coordinates analysis (PCoA) plot based on Bray-Curtis (**a**) or Sorensen-Dice (**b**) dissimilarities, depicting the clusters of bacterial communities grouped according to the facility category. Abbreviations: ECU, Emergency Care Unit; MU, Medical Unit; SC, Surgery Center; IU, Inpatient Unit; ICU-A, Intensive Care Unit A; ICU-B, Intensive Care Unit B.

To explore the relationship among the bacterial communities of all samples, principal coordinates analyses (PCoA) based on Sorensen-Dice and Bray-Curtis indices were performed. Both analyses demonstrated that no pattern of clustering was observed (Figure 2a,b).

To investigate whether the sample grouping in different categories was statistically significant, the non-parametric multivariate statistical test ANOSIM (analysis of similarity) was performed on the distance matrices generated from the beta diversity step. The *p*-value observed indicated that there were significant differences for the four categories. However, the low correlation value of R suggested that the clustering of samples based on the categories was relatively weak (Table S2).

When attempting to correlate the sample grouping with environmental parameters, no significant relationship was observed for surface temperature, ambient temperature, and relative humidity (Table S3).

**Figure 3:**
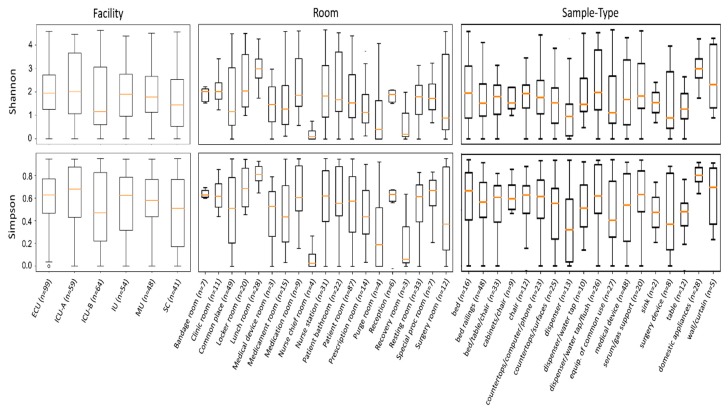
Boxplots showing the distribution of Shannon and Simpson diversity indices for samples grouped in the facility, room, and sample type categories. Abbreviations: ECU, Emergency Care Unit; MU, Medical Unit; SC, Surgery Center; IU, Inpatient Unit; ICU-A, Intensive Care Unit A; ICU-B, Intensive Care Unit B.

We next attempted to study the bacterial diversity in each grouping category using Shannon and Simpson diversity metrics. The results are summarized in Figure 3. For the facility category, no significant difference was observed. For the room and sample type categories, differences were only evident in some cases.

### 2.4. Single OTUs

From the 878 OTUs, 347 (42.6%) were present only once (Table S4). With the exception of *Pseudomonas cremoricolorata*, which is one of the taxonomically and ecologically closely-related species of the Pseudomonas putida species complex, the most prevalent species from these OTUs are usually not associated with the hospital environment nor considered potential pathogens.

**Figure 4:**
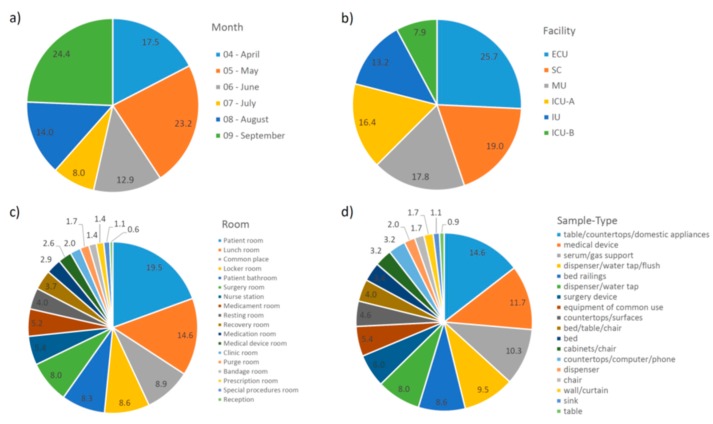
Percentage of single operational taxonomy units (OTUs) in each grouping category. (**a**) Month; (**b**) Facility; (**c**) Room; (**d**) Sample Type. Abbreviations: ECU, Emergency Care Unit; MU, Medical Unit; SC, Surgery Center; IU, Inpatient Unit; ICU-A, Intensive Care Unit A; ICU-B, Intensive Care Unit B.

In order to find any pattern among the single OTUs, their percentages in each grouping category were summarized and plotted in Figure 4. As observed in Figure 4b, the Emergency Care Unit (ECU) was the facility with the highest percentage of OTUs (25.7%), and Intensive Care Unit B (ICU-B) had the lowest (7.9%).

Although 19% of the single OTUs were found in the Surgery Center (SC), only 8% and 6% were found in the surgery room and surgery device, respectively, which indicate that other non-strict SC places, i.e., those also found in other facilities (especially the lunch room, common place, and locker room), have higher concentrations of such transient, sporadic taxa. In other facilities, the prevalence of single OTUs was observed in sample types (Figure 4d) related to a patient room and the lunch room (Figure 4c).

### 2.5. Differential Abundance

The QIIME Python script group_significance.py was used to calculate significant changes, using Kruskal-Wallis analysis as the significance test. Differences were considered significant when Bonferroni adjusted *p*-values < 0.05. The Kruskal-Wallis test identified 12 OTUs showing differential abundances among the facilities (Table 2). Most of them were associated with ICU-B and ICU-A. The presence of nosocomial pathogens was also notable, including *Acinetobacter baumannii*, *Staphylococcus epidermidis*, *Pseudomonas aeruginosa*, *Escherichia coli*, and *Acinetobacter nosocomialis*.

### 2.6. Most Prevalent OTUs Across Samples

**Figure 5:**
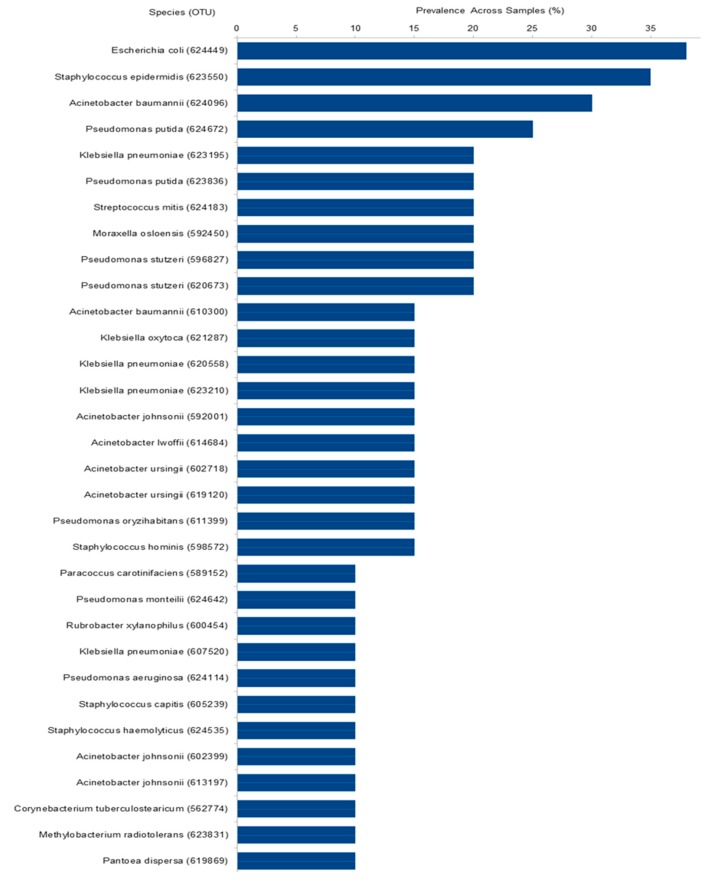
Prevalence of the 32 taxa present in more than 10% of the samples. Axis X represents species’ name followed by OTU number between parentheses.

Out of the total 878 bacterial OTUs identified in the hospital community, 32 were present in more than 10% of the samples (Figure 5), and 70 were present in more than 5% of the samples. The three most abundant were *Escherichia coli*–OTU624449 (38%), *Staphylococcus epidermidis*–OTU623550 (35%), and *Acinetobacter baumannii*–OTU624096 (30%). Here as well, the presence of several nosocomial pathogens was notable. Indeed, 30% of the OTUs present in more than 5% of the samples were composed of nosocomial pathogens and 20% of rare nosocomial pathogens. Only 14% were composed of non-pathogens. The pathogen status of the 70 OTUs is presented in Table S5.

### 2.7. Co-Occurrence Network Analysis

**Figure 6:**
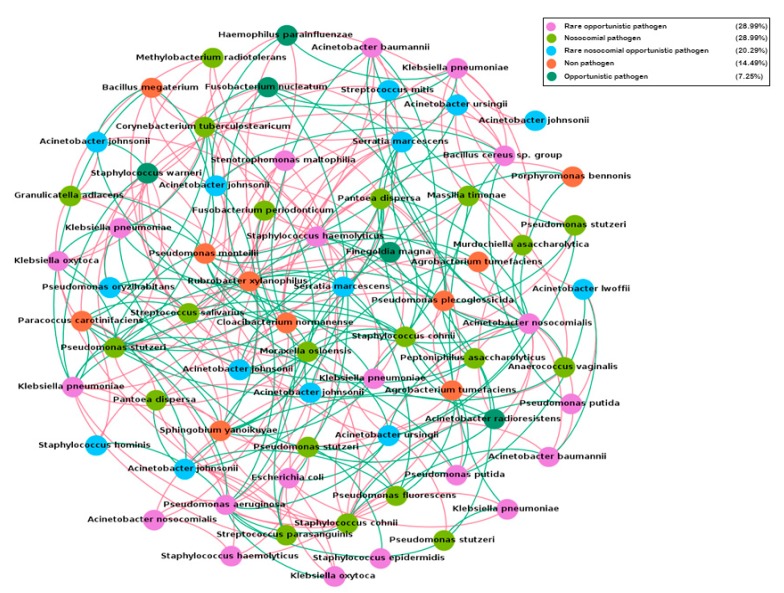
Network analysis of the hospital microbiome present in more than 5% of the samples. Nodes are colored according to the pathogen status of each species (Table S5). Red edges represent negative interactions and green edges represent positive interactions.

The hospital microbial network (Figure 6) consisted of 70 nodes or OTUs (representing the 70 taxa present in more than 5% of the samples) and 274 edges (with a mean of 3.9 edges per node). The clustering coefficient (that is, the extent to which nodes are embedded in their neighborhood) was 0.074 and the modularity index was 0.282 (values >0.4 suggest that the network has a modular structure). Those results indicated that the hospital microbial network had relatively low connectedness, forming a clustered topology, but not structured among groups of nodes (i.e., modules). From the 274 interactions (Table S6), 140 (51.46%) were negative and 134 (48.54%) were positive.

To further explore the clinical relevance of the spatial co-occurrence network from the hospital microbiota, we analyzed the interaction patterns of non-pathogenic species against nosocomial and rare nosocomial pathogens. The results indicated that 43.47% of the interactions were negative, including negative interactions with several nosocomial pathogens like *Acinetobacter nosocomialis*, *Klebsiella pneumoniae*, *Serratia marcescens*, and *Staphylococcus haemolyticus* (Table S6).

To study the influence of potential keystone species within the hospital microbial network, two measures were used: betweenness centrality (which indicates the relevance of a node as capable of holding together communicating nodes) and eigenvector centrality (used to measure the importance of a node by the number of important nodes the node links to). The ranking of OTUs was different for each parameter, which was expected, considering that they were calculated in different ways (Table S5). However, they both had a particular feature, i.e., only the first three OTUs in each rank presented high enough scores to be considered as keystone OTUs. *Acinetobacter nosocomialis*–OTU606882, for example, presented the highest number of interactions (degree: 21) and score for betweeness centrality (148.87), though only prevalent in 5% of the samples.

### 2.8. Pattern of Samples with No Reads

**Figure 7:**
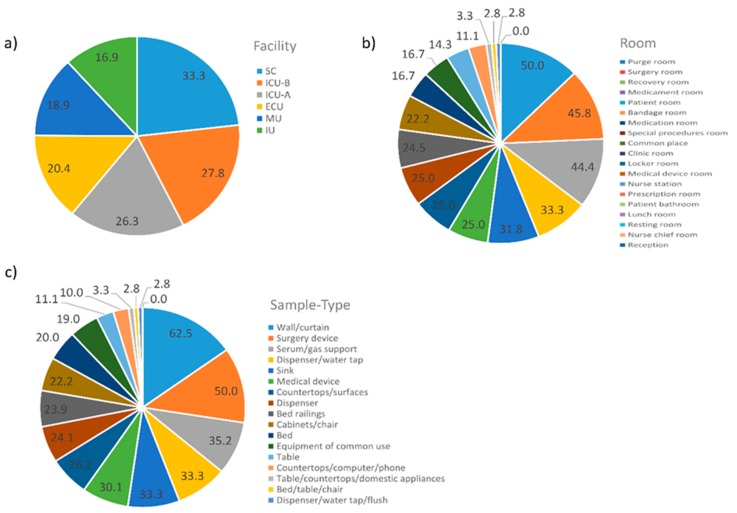
Percentages of the 161 samples with no reads in each one of the three grouping categories. (**a**) Facility; (**b**) Room; (**c**) Sample Type. Abbreviations: ECU, Emergency Care Unit; MU, Medical Unit; SC, Surgery Center; IU, Inpatient Unit; ICU-A, Intensive Care Unit A; ICU-B, Intensive Care Unit B.

Due to the high number of samples with no reads, we tried to find any pattern related to them. In this regard, samples were grouped in different categories. The results are summarized in Figure 7. In terms of facilities, SC (33.3%) followed by both ICU-A (27.8%) and ICU-B (26.3%) presented the highest percentages of samples with no reads. Purge room (50.0%), surgery room (45.8%), recovery room (44.4%), and medication room (33.3%) presented the highest percentages of samples with no reads, while reception (0.0%), nurse chief room (2.8%), resting room (2.8%), and lunch room (3.3%) presented the lowest. Sample types related to these rooms followed the same pattern.

## 3. Discussion

In this study, we used high quality sequences generated from 16S rRNA gene amplicons to explore the bacterial population at inanimate surfaces of different hospital environments and ascertain the accuracy of routine microbiological identification of a broad range of clinically relevant bacterial taxa. To increase the taxonomic profiling and specificity of 16S-based identification, we applied a strict sequence quality filtering process including pre-clustering of our sequences into OTUs at the 100% identity level (with the removal of clusters with less than 5 reads), followed by alignment against a curated reference database at the 99% identity level.

Given our interest in identifying medically relevant taxa, we focused our analysis on OTUs classified at the species level sharing a 99% 16S rRNA gene sequence identity to match bacterial names with standing in nomenclature. In our analysis, 17% of the sequences were not able to be classified at the species level.

Another important aspect of our approach was the primers chosen for the amplification of the 16S rRNA gene. To improve the taxonomic resolution in our analysis, we used the primers 341F and 806R, which generated an amplicon of 465 bp flanking the hypervariable V3-V4 region of the 16S rRNA. This primer set provides ample information for taxonomic classification of microbial communities from specimens associated with human microbiome studies [20]. Therefore, the data generated using the V3-V4 primer pair from sequencing using the MiSeq platform can be compared with the existing data in the human microbiome literature, especially for bacterial skin microbiomes [21], which are of primary relevance for high-touch surface samples. High-touch surfaces are recognized as a possible reservoir of infectious agents and their contamination can also pose a risk for the spread of pathogens [22,23]. In fact, there is now strong evidence from a series of studies and reports that contaminated surfaces contribute to the transmission of hospital pathogens [24]. In a retrospective study, for example, there was more than a two-fold risk of acquiring *Clostridium difficile* if the prior room occupant had this infection [25]. A similar risk was noted for *Acinetobacter* spp. and *Pseudomonas aeruginosa* [26].

But the actual proportion of HAIs attributed to environmental surfaces is largely unknown. This uncertainty occurs mainly because it is complicated to track the transmission of pathogens in healthcare settings and very difficult to link a specific transmission event or infection to an environmental source. Many attempts have been made, but they have been individual [24]. At the hospital level, it is important to have a comprehensive infection prevention program that tracks not only HAIs and major nosocomial pathogens individually, but also all other microorganisms composing that community. Such an approach is essential to assess temporal or geographic patterns that might suggest how microbial taxa spread and interact with each other in the hospital environment, which is of critical importance in determining the role of hospital surfaces and equipment in vectoring pathogen and non-pathogen microbes.

Given the sparse and fragmented knowledge related to the microbial communities present in hospital environments, our comprehensive study was designed to provide a general picture of the hospital microbiome with its diversities and dynamics while being able to focus as well on those taxa of primary relevance for HAIs. Our findings complement the major work of Lax et al. (2017), focused on the bacterial dynamics among hospital surfaces, patients, and staff over the course of 1 year as a new hospital became operational [27]. Overall, the results of beta diversity revealed an overlap among the bacterial communities present in the six facilities, which suggests that hospital microbiomes present a homogeneous structure. Differences in alpha diversity were dependent on individual sample grouping at room and sample type categories. Based on these results, it was possible to answer the two main questions of this work. The answer to the first question, related to potential differences among the microbial communities of the different hospital environments, was contrary to our initial hypothesis that predicted a heterogeneity among the hospital facilities. Such heterogeneity was expected based on previous studies indicating significant differences when two or more hospital facilities were compared, and also by the unique operating features (e.g., type and number of patients and professionals) and cleaning/sterilization routines of each facility, which can generate different selective pressures in each environment.

However, the results of this study indicate that this hospital microbiome is homogeneous. Such homogeneous features of the microbial communities were the basis of the answer to the second question of this work, i.e., whether there was a pattern characterizing the hospital microbiome as a whole. Overall, our findings corroborated the hypothesis that the hospital microbiome presents a characteristic pattern, i.e., with a homogeneous structure composed by a massive dominance of a few taxa and microbial network with low connectedness forming a clustered topology.

In contrast to previous studies suggesting a higher diversity of microbial communities in hospital environments [16,19], our study indicates a massive dominance of a few taxa in all taxonomic levels down to the genera, where the ten most abundant genera in each facility represented 64.4% of all observed taxa. These differences may be partially explained by the removal of 17% of the sequences not classified at the species level for analysis, and, most probably, due to the different and roughly strict pipeline we employed to analyze the 16S rRNA gene high-throughput sequencing data.

While previous pipelines generate high numbers of OTUs (many of them considered spurious, as the result of low quality filtering processes), usually with low taxonomic resolution and ambiguous taxon level identities [17,18,28,29], our analysis resulted in 878 OTUs, in which many of them belonged to the same species. This means that the hospital environment is predominantly inhabited by closely related taxa, a pattern quite different from other environments usually composed of a high diversity even at high taxonomy levels [9,11]. Such patterns also explain the results in the PCoA analysis, which indicated that the structure of the bacterial communities in the hospital environments were similar.

In this sense, closely related taxa may play similar roles within the hospital, while their distributions may vary significantly in each environment. The closely related species *Acinetobacter pitti* and *Acinetobacter nosocomialis*, for example, may play a role similar to *Acinetobacter baumannii*, but the relative distribution of these three Acb complex species seem to vary geographically [30]. Notably, we observed a high presence of several potential nosocomial pathogens, including *Acinetobacter baumannii*, *Acinetobacter nosocomialis*, *Bacillus cereus*, *Klebsiella oxytoca*, *Pseudomonas aeruginosa*, *Pseudomonas putida*, *Staphylococcus aureus*, *Staphylococcus epidermidis*, and *Serratia marcescens*, among others. Some of these nosocomial species were also differentially abundant in the hospital facilities analyzed, which may provide better clues about the preferential habitats of these particular species, as well as their potential reservoirs in hospital environments. The presence of potential pathogens differentially abundant in ICU-B and ICU-A may be explained by the fact that these facilities usually receive patients with severe illnesses, some of them associated with severe infections caused by these pathogens. Nevertheless, their presence in inanimate surfaces should be of primary concern and demonstrate that the cleaning routine of these environments should be reviewed.

The pattern of single OTUs gives better clues on the main routes of entry of microorganisms in the hospital environment. Those samples and sites with higher concentrations of transient, sporadic taxa, are probably the ones disseminating new microorganisms within hospitals. In our study, a higher prevalence of single OTUs was observed in samples related to patient room, lunch room, common place, and locker room.

The pattern of samples with no reads also provides an expected, but noteworthy feature of the hospital microbiome, i.e., the highest percentage of samples with no reads was usually associated with samples known to undergo rigorous cleaning and sterilization procedures, for example, SC (33.3%), ICU-A (27.8%), and ICU-B (26.3%), in terms of facility. On the other hand, the lowest percentage of samples with no reads was usually associated with samples known to undergo less rigorous cleaning procedures; e.g., reception (0.0%), nurse chief room (2.8%), resting room (2.8%), and lunch room (3.3%), in terms of rooms.

By employing network analyses, we described the complex pattern of inter-relationships between bacterial taxa co-occurring in the hospital environment. Only correlations with r ≥ ±0.9 (p ≤ 0.05) were used to generate the hospital network. Such strict cutoff increases the confidence of our analysis for detecting only strong interactions, which ensures that strongly non-random distribution patterns are mostly due to ecological reasons. Positive correlations suggest the occurrence of a mutualistic interaction while negative correlations suggest the presence of direct or indirect competition between the bacterial taxa co-occurring in the hospital environment.

The results indicated that the hospital microbial network presents a unique co-occurrence pattern by forming a low connectedness and clustered topology, but was not structured among groups of nodes (i.e., modules), as usually found in most natural environments [31,32]. These structural properties offer the potential for comparison among different healthcare ecosystems in order to explore how the general traits of a given hospital may influence the assembly of microbial communities. It also helps us understand which organisms are most important in maintaining the structure and interactions of microbial communities in hospital facilities. In this sense, the identification of potential keystone species is of primary relevance. A keystone species is a taxon whose importance is relatively higher than others for maintaining the structure of a community. [33]. In this study, the two most suitable parameters (defined by Rampelotto et al., 2014 [34]) identified only a few potential keystone taxa, which suggests that the hospital microbial network is evenly distributed.

A novel and important strength of our co-occurrence analysis is the ability to detect ecologically relevant relationships between specific microbial taxa, in particular, potential competition between pathogens and non-pathogens. To date, a limited number of studies addressing the events associated with microbial competition in a spatial and multiplexed fashion have been performed, due in part to the lack of available tools. In this sense, we hypothesize that this strategy could be used as a theoretical framework to identify potentially strong negative correlations between pathogens and non-pathogens, which in turn could guide more focused and experimental studies to screen for bioactive compounds against pathogenic bacteria derived from any non-pathogenic microbe that possesses a competitive advantage. As a proof of concept, by using in vitro co-cultivation, Gonzalez et al., 2011 demonstrated that the non-pathogenic *Bacillus subtilis*, a bacterium that is nearly ubiquitous in nature, was able to inhibit the growth of an epidemic *Staphylococcus aureus* isolate and possessed the ability to directionally release a molecule with antimicrobial and metabolism-altering properties [35]. In another interesting case, a recent in vitro study provided the first evidence that the harmless bacteria *Corynebacterium accolens*, which commonly colonizes the nose, can help inhibit *Streptococcus pneumoniae* through a direct antagonistic interaction between these species [36].

In our study, we have identified different taxa presenting a strong negative co-occurrence with a variety of nosocomial and rare nosocomial pathogens. These taxa could be the focus of future in vitro co-culture experiments exploring the underlying mechanisms of antagonistic interactions between commensal and pathogenic bacteria, to address how one species prevents the growth of another and to identify which components are involved in such interactions.

Another promising application would be the direct use of the non-pathogenic species or genetically engineered harmless variants of rare opportunistic pathogens as microbial-based sanitizing agents to reduce and control the colonization of nosocomial pathogens. This new concept, originally suggested by Falagas and Makris [37], has already successfully been applied in recent years as an alternative method to chemical disinfectants [38,39,40]. The rational design and use of probiotic bacteria and biosurfactants for nosocomial infection control may overcome the problems associated with the chemical germicides, which present risks towards the environment and the patient’s safety [41]. Several studies have demonstrated that more than 50% of hospital room surfaces are inadequately cleaned and disinfected when conventional chemical disinfectants are used. In addition, disinfectants can select resistant bacterial strains against themselves and also against antibiotics [42], which has been recently reported for chlorhexidine induction of resistance against Colistin [43], an antibiotic considered, until 2016, as a last-resort drug for treatment of infections sustained by multidrug-resistant (MDR) gram-negative bacteria.

This emerging concept of microbial remediation for the prevention and control of hospital-acquired infections is a paradigm shift in the field, in which, instead of eradicating all pathogens, replacing pathogens by beneficial microbes might be more effective in decreasing infections [44,45,46].

Although 16S rRNA gene sequencing as a clinical screening tool has many advantages over traditional culture-based techniques, it is important to ponder its limitations. As any amplification based on rRNA genes, it only analyzes a short, specific genomic region, and taxonomic resolution or functional inference may be limited, especially for closely related species (i.e., sharing >99% similarity in their 16S rRNA gene sequence) [47,48,49]. As an example, *Clostridium botulinum* and *Clostridium sporogenes* exhibit a 99.7% similarity [50,51]. Another example may be found in the genus *Rickettsia*, in which 16S rRNA gene sequence similarity values >99% are found among all 26 species that have names with standing in nomenclature [52]. In general, 2.4% of complete sequenced genomes have 16S rRNA sequences with <99% mean similarities [53]. Another major limitation of the 16S rRNA sequence is its inability to discriminate among virulent strains, which means it is not possible to distinguish pathogenic *Clostridium difficile* or *Escherichia coli* strains from nonpathogenic strains. Thus, for accurate identification of certain bacterial species and virulent strains, further methods, such as multiplex PCR assay, mass spectrometry, or whole genome sequencing must be applied.

Despite these limitations, the framework provided in this study for the detection of multiple clinically relevant microbial targets is a promising addition to current diagnostic techniques and can play an important role in routine healthcare-associated infections’ surveillance.

## 4. Materials and Methods

### 4.1. Sampling Site and Collection

The study was carried out at a tertiary-level teaching hospital with 200 beds located in southern Brazil. 111 surface samples were collected monthly at six hospital facilities between April–September 2015, including two intensive care units (ICU-A and ICU-B), one surgery Ccenter (SC), one medical unit (MU), one inpatient unit (IU), and one emergency care unit (ECU). In total, 663 samples were collected during the six-month period of the study (three samples were discarded due to problems during the sequencing). The types of surfaces sampled within the facilities were chosen based on the frequency with which the surfaces were touched (here defined as high-touch surfaces), such as workstations, medical, and surgical devices. All sampling locations and their characteristics are given in Supplementary Table S1. Beyond individual characterization, samples were also grouped in four categories, named month, facility, room, and sample type. For example, all samples collected in April were grouped in the “April” month category, all samples collected in the emergency care unit during the six-month period of sampling were grouped in the “ECU” facility category. Room and sample type categories followed the same principle.

Sterile swabs and gloves were used for sampling collection. Swabs were moistened with sterile saline solution and streaked across the surface of each sample. After sampling, the swabs were transported back to the laboratory for DNA extraction, library preparation, and DNA sequencing.

### 4.2. Environment Measurements

Measurements of relative humidity and air temperature were carried out soon after the sample collection using a digital hygro-thermometer (Incoterm–TTH100). For surface temperature measurements, a digital laser infrared thermometer (GM300, Benetech) was used.

### 4.3. DNA Extraction, PCR Amplification, and Amplicon Sequencing

DNA was extracted following an optimized magnetic bead-based DNA extraction and purification protocol, owned by Neoprospecta Microbiome Technologies (Brazil). Barcoded PCR amplification was performed using the 341F and 806R primers (with 465 bp amplicons flanking the highly variable V3-V4 region of the 16S rRNA gene sequence) with the following conditions: the first PCR primers contain the Illumina sequences based on the TruSeq structure adapter (Illumina, San Diego, CA, USA), allowing the second PCR with indexing sequences. PCR was always carried out in triplicate using Platinum Taq (Invitrogen, USA) with the conditions: 95 °C for 5 min, 25 cycles of 95 °C for 45 s, 55 °C for 30 s, 72 °C for 45 s, and a final extension of 72 °C for 2 min for PCR 1. In PCR 2 the conditions were 95 °C for 5 min, 10 cycles of 95 °C for 45 s, 66 °C for 30 s, 72 °C for 45 s, and a final extension of 72 °C for 2 min. Taq Platinum was chosen due to its capacity to better amplify samples with low amounts of DNA (i.e., <5 ng) and PCR cycles for the amplicon PCR were reduced to 21 to diminish PCR bias. The final PCR was cleaned up using AMPureXP beads (Beckman Coulter, Brea, CA, USA) and samples were pooled in the sequencing libraries for quantification. Library estimations were performed with Picogreen dsDNA assays (Invitrogen, USA), and then the libraries were diluted for accurate quantification by qPCR using the KAPA library quantification kit for Illumina platforms (KAPA Biosystems, Woburn, MA, USA). The libraries were sequenced in a MiSeq system using a V2 kit, with a single-end 300 nt run.

### 4.4. 16S rRNA Reads Processing for Downstream Analyses

Sequencing raw data from MiSeq was processed using a customized python script. Briefly, all the reads were individually submitted to a quality filter, based on the sum of the DNA bases’ probabilities errors, allowing a maximum of 1% of accumulated errors. Subsequently, the DNA sequences corresponding to the Illumina adapters were removed. Sequences that presented 100% identity were clustered and defined as an operational taxonomy unit (OTU). If any cluster was represented by fewer than 5 reads, it was not considered in further analysis. Each OTU was then aligned against a private reference alignment database (owned by Neoprospecta) at the 99% identity level, using Blast [54]. The taxonomy associated with each OTU was assigned as the taxonomy associated with the reference sequence defining the OTU. For all OTU-based analyses except the co-occurrence network, the original OTU table was normalized using cumulative sum scaling (CSS) method [55].

### 4.5. Community Composition and Diversity Analysis

QIIME version 1.9.0 was used to estimate alpha and beta diversity [56]. OTU abundances were used to calculate the alpha diversity metrics, including OTU richness (unique OTUs), ChaoI richness estimation and Shannon’s diversity indices. For overall comparison of significant differences among bacterial communities (i.e., beta diversity), principal coordinates analysis (PCoA) was performed. Samples were grouped in four categories, named month, facility, room, and sample type (Table S1). A matrix using Bray-Curtis and Sørensen-Dice metrics for each pair of environments was calculated. The distances were turned into points in space with the number of dimensions one less than the number of samples. The first three principal dimensions were used to plot a three-dimensional graph that was visualized using EMPeror [57].

To achieve statistical confidence for the sample grouping observed by PCoA (month, facility, room, and sample type), we performed the ANOSIM multivariate test, using the vegan package, through the compare_category.py script of QIIME. The otu_category_significance.py script was run using the ANOVA to find OTUs whose members were differentially represented among the hospital facilities. Moreover, to analyze whether there was any significant relationship between samples and environmental parameters, we performed the Mantel test. The most prevalent OTUs across samples were analyzed with compute_core_microbiome.py at different cut-off values.

### 4.6. Co-Occurrence Network Analysis

Non-random co-occurrence network analyses were performed using SparCC from the raw count OTU table [58]. Ten interactions were used to estimate the median correlation of each pairwise and the statistical significance of the correlations was calculated by bootstrapping with 100 iterations. SparCC correlations with a magnitude of 0.9 and statistical significance (*p* < 0.01) were incorporated into the network analyses. The nodes in the reconstructed networks represent the OTUs, whereas the edges (that is, connections) correspond to a strong and significant (positive or negative) correlation between nodes. In order to describe the topology of the resulting network, two centrality measures (i.e., betweenness centrality and eigenvector centrality) were calculated and the network was visualized using the interactive platform Gephi [59].

## 5. Conclusions

The results of our investigation provide new insights into different aspects of the hospital microbiome and indicate that the high-throughput sequencing of the 16S rRNA gene can be used as a robust first-step tool for microbiological identification and characterization of a wide range of common bacterial pathogens in hospital settings with high resolution. Through the use of a well-annotated database of 16S rRNA sequences, and the use of a rigorous filtering process, we have demonstrated that high-resolution profiling of bacterial communities can be achieved and have concluded that the framework developed in this study may be an integral part of routine diagnostic testing for hospital surveillance and infection control. Further improvements on the framework can make this technology an even more user-friendly tool in the routine of hospitals, to control, and more importantly, prevent, hospital outbreaks. This approach also shows potential for clinical application in infectious disease diagnostics, an area in which 16S rRNA gene sequence identification might have an immediate and direct impact on patient care.

## Figures and Tables

**Table 1:** Most abundant genera in the hospital facilities. Relative abundances are shown as percentages (%). Abbreviations: ECU, Emergency Care Unit; MU, Medical Unit; SC, Surgery Center; IU, Inpatient Unit; ICU-A, Intensive Care Unit A; ICU-B, Intensive Care Unit B.

Phyla	Genera	Total	ICU-B	ICU-A	IU	MU	ECU	SC
Proteobacteria	*Acinetobacter*	17.2	17.8	15.9	18.1	14.9	21.6	15.0
Proteobacteria	*Pseudomonas*	16.2	17.9	16.7	13.1	16.5	18.4	14.4
Firmicutes	*Staphylococcus*	6.8	9.0	8.6	6.4	5.9	3.5	7.2
Proteobacteria	*Klebsiella*	6.6	8.5	9.0	6.5	7.2	2.2	6.4
Firmicutes	*Streptococcus*	3.5	2.3	3.8	3.5	4.5	1.4	5.7
Proteobacteria	*Pantoea*	3.4	3.5	4.9	5.0	1.0	3.4	2.9
Firmicutes	*Bacillus*	3.3	1.2	2.0	3.7	5.5	6.0	1.6
Proteobacteria	*Escherichia*	2.9	4.7	3.3	2.4	2.3	2.2	2.8
Proteobacteria	*Stenotrophomonas*	2.3	1.7	1.7	2.7	2.8	3.6	1.5
Proteobacteria	*Moraxella*	2.2	1.7	1.7	2.5	1.7	3.1	2.5

**Table 2:** Differentially abundant operational taxonomy units (OTUs) in each hospital facility. Statistical confidence was accessed using *the Krustal-Wallis test*.

OTU	Species	Bonferroni p	ICU-B	ICU-A	IU	MU	ECU	SC
596827	*Pseudomonas stutzeri*	1.78 × 10^−10^	4.41	2.34	0.55	2.92	0.51	1.20
624096	*Acinetobacter baumannii*	7.00 × 10^−6^	4.50	4.42	1.87	3.65	1.22	3.07
623550	*Staphylococcus epidermidis*	2.72 × 10^−5^	4.66	4.12	3.14	3.30	1.25	4.13
602646	*Aerococcus viridans*	0.01	0.09	0.30	0.40	0.11	0.23	1.48
617614	*Anaerococcus vaginalis*	0.01	1.33	0.74	0.00	0.00	0.44	0.13
587128	*Murdochiella asaccharolytica*	0.01	1.30	0.57	0.00	0.00	0.34	0.24
584857	*Porphyromonas bennonis*	0.01	1.30	0.85	0.00	0.00	0.41	0.12
624114	*Pseudomonas aeruginosa*	0.01	2.35	0.99	0.48	1.52	0.32	0.92
614684	*Acinetobacter lwoffii*	0.01	1.85	1.15	0.97	1.17	3.23	0.35
624449	*Escherichia coli*	0.01	5.46	4.37	2.92	3.22	2.21	3.92
598572	*Staphylococcus hominis*	0.01	2.38	1.96	1.30	0.62	0.54	0.99
607150	*Acinetobacter nosocomialis*	0.02	1.36	1.02	0.00	0.00	0.66	0.25

## Supplementary Materials

Supplementary materials can be found at https://www.mdpi.com/1422-0067/20/12/3099/s1.

## Abbreviations

CDC	Centers for Disease Control and Prevention
ECU	Emergency Care Unit
HAIs	Hospital-Acquired Infections
ICU-A	Intensive Care Unit A
ICU-B	Intensive Care Unit B
IU	Inpatient Unit
MU	Medical Unit
OTU	Operational Taxonomy Unit
PCoA	Principal Coordinates Analysis
SC	Surgery Center
