# Supplementary material for: Exploring the Hospital Microbiome by High-Resolution 16S rRNA Profiling
Source: Int J Mol Sci. 2019 Jun 25;20(12):3099. doi: 10.3390/ijms20123099 (PMC6696720; doi:10.3390/ijms20123099)
Supplement: Supplementary file 1 [file ijms-20-03099-s001.pdf]

Table S1. Library characterization. Month, Facility, Room, and Sample-Type represent the four categories which samples were grouped. (%). Abbreviations: ECU, Emergency Care Unit; MU, Medical Unit; SC, Surgery Center; IU, Inpatient Unit; ICU-A, Intensive Care Unit A; ICU-B, Intensive Care Unit B.

Sample Code	Sample Description	Sample-Type	Room	Facility	Month
150410886613.1.1	Dispenser, water tap and flush from Patient Bathroom A	dispenser/water tap/flush	Patient bathroom	IU	04 - April
150410886614.1.1	Serum and gas support from Patient Room 1	serum/gas support	Patient room	IU	04 - April
150410886615.1.1	Bed railings from Patient Room A	bed railings	Patient room	IU	04 - April
150410886616.1.1	Dispenser, water tap and flush from Patient Bathroom B	dispenser/water tap/flush	Patient bathroom	IU	04 - April
150410886617.1.1	Bed railings from Patient Room B	bed railings	Patient room	IU	04 - April
150410886618.1.1	Serum and gas support from Patient Room 2	serum/gas support	Patient room	IU	04 - April
150410886619.1.1	Countertops, computer and phone	countertops/computer/phone	Nurse station	IU	04 - April
150410886620.1.1	Dispenser and water tap	dispenser/water tap	Medicament room	IU	04 - April
150410886621.1.1	Countertops and surfaces	countertops/surfaces	Medicament room	IU	04 - April
150410886622.1.1	Countertops and surfaces	countertops/surfaces	Bandage room	IU	04 - April
150410886623.1.1	Table, countertops and domestic appliances	table/countertops/domestic appliances	Lunch room	IU	04 - April
150410886624.1.1	Bed, table and chair	bed/table/chair	Resting room	IU	04 - April
150410886625.1.1	Dispenser from the Hall	dispenser	Common place	IU	04 - April
150410886626.1.1	Glicosimeter	medical device	Common place	IU	04 - April
150410886641.1.1	Dispenser, water tap and flush from Patient Bathroom A	dispenser/water tap/flush	Patient bathroom	MU	04 - April
150410886642.1.1	Bed railings from Patient Room A	bed railings	Patient room	MU	04 - April
150410886643.1.1	Serum and gas support from Patient Room 1	serum/gas support	Patient room	MU	04 - April
150410886644.1.1	Dispenser, water tap and flush from Patient Bathroom B	dispenser/water tap/flush	Patient bathroom	MU	04 - April

150410886645.1.1	Bed railings from Patient Room B	bed railings	Patient room	MU	04 - April
150410886646.1.1	Serum and gas support from Patient Room 2	serum/gas support	Patient room	MU	04 - April
150410886647.1.1	Countertops, computer and phone	countertops/computer/phone	Nurse station	MU	04 - April
150410886648.1.1	Dispenser and water tap	dispenser/water tap	Medicament room	MU	04 - April
150410886649.1.1	Countertops and surfaces	countertops/surfaces	Medicament room	MU	04 - April
150410886650.1.1	Cabinets and chairs	cabinets/chair	Locker room	MU	04 - April
150410886651.1.1	Dispenser and water tap	dispenser/water tap	Locker room	MU	04 - April
150410886652.1.1	Table, countertops and domestic appliances	table/countertops/domestic appliances	Lunch room	MU	04 - April
150410886653.1.1	Bed, table and chair	bed/table/chair	Resting room	MU	04 - April
150410886654.1.1	Dispenser from the Hall	dispenser	Common place	MU	04 - April
150410886655.1.1	Glicosimeter	medical device	Common place	MU	04 - April
150410886664.1.1	Bed railings	bed railings	Recovery room	SC	04 - April
150410886665.1.1	Serum and gas support	serum/gas support	Recovery room	SC	04 - April
150410886666.1.1	Countertops and surfaces	countertops/surfaces	Recovery room	SC	04 - April
150410886667.1.1	Surgical table from Room 1	bed	Surgery room	SC	04 - April
150410886668.1.1	Oxigen buttons	surgery device	Surgery room	SC	04 - April
150410886669.1.1	Laryngoscope instrument tray	surgery device	Surgery room	SC	04 - April
150410886670.1.1	Anesthesia syringes	surgery device	Surgery room	SC	04 - April
150410886671.1.1	Surgical table from Room 2	bed	Surgery room	SC	04 - April
150410886672.1.1	Surgical light	surgery device	Surgery room	SC	04 - April
150410886673.1.1	Syringes	surgery device	Surgery room	SC	04 - April
150410886674.1.1	Electric scalpel	surgery device	Surgery room	SC	04 - April
150410886675.1.1	Cabinets and chairs	cabinets/chair	Locker room	SC	04 - April
150410886676.1.1	Dispenser and water tap	dispenser/water tap	Locker room	SC	04 - April
150410886677.1.1	Bed, table and chair	bed/table/chair	Resting room	SC	04 - April
150410886678.1.1	Table, countertops and domestic appliances	table/countertops/domestic appliances	Lunch room	SC	04 - April
150410886679.1.1	Computer	equipment of common use	Nurse chief room	SC	04 - April
150410886680.1.1	Stretcher from the Hall	bed	Common place	SC	04 - April

150410886681.1.1	Dispenser from the Hall	dispenser	Common place	SC	04 - April
150410886696.1.1	Bed railings from Patient Room A	bed railings	Patient room	ICU-A	04 - April
150410886697.1.1	Infusion pumps	medical device	Patient room	ICU-A	04 - April
150410886698.1.1	Screen monitors	medical device	Patient room	ICU-A	04 - April
150410886699.1.1	Curtain	wall/curtain	Patient room	ICU-A	04 - April
150410886700.1.1	Bed railings from Patient Room B	bed railings	Patient room	ICU-A	04 - April
150410886701.1.1	Utensils table	table	Patient room	ICU-A	04 - April
150410886702.1.1	Wall	wall/curtain	Patient room	ICU-A	04 - April
150410886703.1.1	Termometer	medical device	Patient room	ICU-A	04 - April
150410886704.1.1	Countertops, computer and phone	countertops/computer/phone	Nurse station	ICU-A	04 - April
150410886705.1.1	Clipboards	equipment of common use	Nurse station	ICU-A	04 - April
150410886706.1.1	Computers	equipment of common use	Prescription room	ICU-A	04 - April
150410886707.1.1	SEquipMateriais - EquipLimpos	medical device	Medical device room	ICU-A	04 - April
150410886708.1.1	Cabinets and chairs	cabinets/chair	Locker room	ICU-A	04 - April
150410886709.1.1	Dispenser, water tap and flush from Bathroom	dispenser/water tap/flush	Locker room	ICU-A	04 - April
150410886710.1.1	Countertops and surfaces	countertops/surfaces	Purge room	ICU-A	04 - April
150410886711.1.1	Dryer	equipment of common use	Purge room	ICU-A	04 - April
150410886712.1.1	Bed, table and chair	bed/table/chair	Resting room	ICU-A	04 - April
150410886713.1.1	Bed, table and chair	bed/table/chair	Resting room	ICU-A	04 - April
150410886714.1.1	Table, countertops and domestic appliances	table/countertops/domestic appliances	Lunch room	ICU-A	04 - April
150410886727.1.1	Bed railings from Patient Room A	bed railings	Patient room	ICU-B	04 - April
150410886728.1.1	Infusion pumps	medical device	Patient room	ICU-B	04 - April
150410886729.1.1	Screen monitors	medical device	Patient room	ICU-B	04 - April
150410886730.1.1	Curtain	wall/curtain	Patient room	ICU-B	04 - April
150410886731.1.1	Bed railings from Patient Room B	bed railings	Patient room	ICU-B	04 - April
150410886732.1.1	Utensils table	table	Patient room	ICU-B	04 - April
150410886733.1.1	Wall	wall/curtain	Patient room	ICU-B	04 - April
150410886734.1.1	Termometer	medical device	Patient room	ICU-B	04 - April

150410886735.1.1	Countertops, computer and phone	countertops/computer/phone	Nurse station	ICU-B	04 - April
150410886736.1.1	Refrigerator	equipment of common use	Nurse station	ICU-B	04 - April
150410886737.1.1	Computers	equipment of common use	Prescription room	ICU-B	04 - April
150410886738.1.1	Dispenser from the Hall	dispenser	Common place	ICU-B	04 - April
150410886739.1.1	Utensils from the Bath cart	medical device	Common place	ICU-B	04 - April
150410886740.1.1	Glicosimeter	medical device	Common place	ICU-B	04 - April
150410886741.1.1	Cufometer	medical device	Common place	ICU-B	04 - April
150410886742.1.1	Bath cart	medical device	Common place	ICU-B	04 - April
150410886743.1.1	Emergency cart	medical device	Common place	ICU-B	04 - April
150410886744.1.1	X-Raio cart	medical device	Common place	ICU-B	04 - April
150410886745.1.1	Ultrasound cart	medical device	Common place	ICU-B	04 - April
150410886746.1.1	Passant	medical device	Common place	ICU-B	04 - April
150410886747.1.1	Physiotherapeutic armchair	medical device	Common place	ICU-B	04 - April
150410886765.1.1	Bed railings from Patient Room A	bed railings	Patient room	ECU	04 - April
150410886766.1.1	Serum and gas support from Patient Room 1	serum/gas support	Patient room	ECU	04 - April
150410886767.1.1	Bed railings from Patient Room B	bed railings	Patient room	ECU	04 - April
150410886768.1.1	Serum and gas support from Patient Room 2	serum/gas support	Patient room	ECU	04 - April
150410886769.1.1	Bed railings from Patient Room C	bed railings	Patient room	ECU	04 - April
150410886770.1.1	Serum and gas support from Patient Room 3	serum/gas support	Patient room	ECU	04 - April
150410886771.1.1	Dispenser, water tap and flush from Patient Bathroom	dispenser/water tap/flush	Patient bathroom	ECU	04 - April
150410886772.1.1	Countertops, computer and phone	countertops/computer/phone	Nurse station	ECU	04 - April
150410886773.1.1	Dispenser and water tap	dispenser/water tap	Medicament room	ECU	04 - April
150410886774.1.1	Countertops and surfaces	countertops/surfaces	Medicament room	ECU	04 - April
150410886775.1.1	Chairs	chair	Medication room	ECU	04 - April
150410886776.1.1	Serum and gas support	serum/gas support	Medication room	ECU	04 - April
150410886777.1.1	Computers	equipment of common use	Prescription room	ECU	04 - April
150410886778.1.1	Sink	sink	Special procedures room	ECU	04 - April

150410886779.1.1	SProcedEspeciais - Leito	bed	Special procedures room	ECU	04 - April
150410886780.1.1	Countertops and surfaces	countertops/surfaces	Bandage room	ECU	04 - April
150410886781.1.1	Bed	bed	Clinic room	ECU	04 - April
150410886782.1.1	Computer table	table	Clinic room	ECU	04 - April
150410886783.1.1	Dispenser and water tap	dispenser/water tap	Clinic room	ECU	04 - April
150410886784.1.1	Chairs	chair	Reception	ECU	04 - April
150410886785.1.1	Table, countertops and domestic appliances	table/countertops/domestic appliances	Lunch room	ECU	04 - April
150410886786.1.1	Bed, table and chair	bed/table/chair	Resting room	ECU	04 - April
150410886787.1.1	Dispenser from the Hall	dispenser	Common place	ECU	04 - April
150410886788.1.1	Medication cart	medical device	Common place	ECU	04 - April
150501772314.1.1	Dispenser, water tap and flush from Patient Bathroom A	dispenser/water tap/flush	Patient bathroom	IU	05 - May
150501772315.1.1	Serum and gas support from Patient Room 1	serum/gas support	Patient room	IU	05 - May
150501772316.1.1	Bed railings from Patient Room A	bed railings	Patient room	IU	05 - May
150501772317.1.1	Dispenser, water tap and flush from Patient Bathroom B	dispenser/water tap/flush	Patient bathroom	IU	05 - May
150501772318.1.1	Bed railings from Patient Room B	bed railings	Patient room	IU	05 - May
150501772319.1.1	Serum and gas support from Patient Room 2	serum/gas support	Patient room	IU	05 - May
150501772320.1.1	Countertops, computer and phone	countertops/computer/phone	Nurse station	IU	05 - May
150501772321.1.1	Dispenser and water tap	dispenser/water tap	Medicament room	IU	05 - May
150501772322.1.1	Countertops and surfaces	countertops/surfaces	Medicament room	IU	05 - May
150501772323.1.1	Countertops and surfaces	countertops/surfaces	Bandage room	IU	05 - May
150501772324.1.1	Table, countertops and domestic appliances	table/countertops/domestic appliances	Lunch room	IU	05 - May
150501772325.1.1	Bed, table and chair	bed/table/chair	Resting room	IU	05 - May
150501772326.1.1	Dispenser from the Hall	dispenser	Common place	IU	05 - May
150501772327.1.1	Glicosimeter	medical device	Common place	IU	05 - May
150501772342.1.1	Dispenser, water tap and flush from Patient Bathroom A	dispenser/water tap/flush	Patient bathroom	MU	05 - May

150501772343.1.1	Bed railings from Patient Room A	bed railings	Patient room	MU	05 - May
150501772344.1.1	Serum and gas support from Patient Room 1	serum/gas support	Patient room	MU	05 - May
150501772345.1.1	Dispenser, water tap and flush from Patient Bathroom B	dispenser/water tap/flush	Patient bathroom	MU	05 - May
150501772346.1.1	Bed railings from Patient Room B	bed railings	Patient room	MU	05 - May
150501772347.1.1	Serum and gas support from Patient Room 2	serum/gas support	Patient room	MU	05 - May
150501772348.1.1	Countertops, computer and phone	countertops/computer/phone	Nurse station	MU	05 - May
150501772349.1.1	Dispenser and water tap	dispenser/water tap	Medicament room	MU	05 - May
150501772350.1.1	Countertops and surfaces	countertops/surfaces	Medicament room	MU	05 - May
150501772351.1.1	Cabinets and chairs	cabinets/chair	Locker room	MU	05 - May
150501772352.1.1	Dispenser and water tap	dispenser/water tap	Locker room	MU	05 - May
150501772353.1.1	Table, countertops and domestic appliances	table/countertops/domestic appliances	Lunch room	MU	05 - May
150501772354.1.1	Bed, table and chair	bed/table/chair	Resting room	MU	05 - May
150501772355.1.1	Dispenser from the Hall	dispenser	Common place	MU	05 - May
150501772356.1.1	Glicosimeter	medical device	Common place	MU	05 - May
150501772365.1.1	Bed railings	bed railings	Recovery room	SC	05 - May
150501772366.1.1	Serum and gas support	serum/gas support	Recovery room	SC	05 - May
150501772367.1.1	Countertops and surfaces	countertops/surfaces	Recovery room	SC	05 - May
150501772368.1.1	Surgical table from Room 1	bed	Surgery room	SC	05 - May
150501772369.1.1	Oxigen buttons	surgery device	Surgery room	SC	05 - May
150501772370.1.1	Laryngoscope instrument tray	surgery device	Surgery room	SC	05 - May
150501772371.1.1	Anesthesia syringes	surgery device	Surgery room	SC	05 - May
150501772372.1.1	Surgical table from Room 2	bed	Surgery room	SC	05 - May
150501772373.1.1	Surgical light	surgery device	Surgery room	SC	05 - May
150501772374.1.1	Syringes	surgery device	Surgery room	SC	05 - May
150501772375.1.1	Electric scalpel	surgery device	Surgery room	SC	05 - May
150501772376.1.1	Cabinets and chairs	cabinets/chair	Locker room	SC	05 - May
150501772377.1.1	Dispenser and water tap	dispenser/water tap	Locker room	SC	05 - May

150501772378.1.1	Bed, table and chair	bed/table/chair	Resting room	SC	05 - May
150501772379.1.1	Table, countertops and domestic appliances	table/countertops/domestic appliances	Lunch room	SC	05 - May
150501772380.1.1	Computer	equipment of common use	Nurse chief room	SC	05 - May
150501772381.1.1	Stretcher from the Hall	bed	Common place	SC	05 - May
150501772382.1.1	Dispenser from the Hall	dispenser	Common place	SC	05 - May
150501772397.1.1	Bed railings from Patient Room A	bed railings	Patient room	ICU-A	05 - May
150501772398.1.1	Infusion pumps	medical device	Patient room	ICU-A	05 - May
150501772399.1.1	Screen monitors	medical device	Patient room	ICU-A	05 - May
150501772400.1.1	Curtain	wall/curtain	Patient room	ICU-A	05 - May
150501772401.1.1	Bed railings from Patient Room B	bed railings	Patient room	ICU-A	05 - May
150501772402.1.1	Utensils table	table	Patient room	ICU-A	05 - May
150501772403.1.1	Wall	wall/curtain	Patient room	ICU-A	05 - May
150501772404.1.1	Termometer	medical device	Patient room	ICU-A	05 - May
150501772405.1.1	Countertops, computer and phone	countertops/computer/phone	Nurse station	ICU-A	05 - May
150501772406.1.1	Clipboards	equipment of common use	Nurse station	ICU-A	05 - May
150501772407.1.1	Computers	equipment of common use	Prescription room	ICU-A	05 - May
150501772408.1.1	SEquipMateriais - EquipLimpos	medical device	Medical device room	ICU-A	05 - May
150501772409.1.1	Cabinets and chairs	cabinets/chair	Locker room	ICU-A	05 - May
150501772410.1.1	Dispenser, water tap and flush from Bathroom	dispenser/water tap/flush	Locker room	ICU-A	05 - May
150501772411.1.1	Countertops and surfaces	countertops/surfaces	Purge room	ICU-A	05 - May
150501772412.1.1	Dryer	equipment of common use	Purge room	ICU-A	05 - May
150501772413.1.1	Bed, table and chair	bed/table/chair	Resting room	ICU-A	05 - May
150501772414.1.1	Bed, table and chair	bed/table/chair	Resting room	ICU-A	05 - May
150501772415.1.1	Table, countertops and domestic appliances	table/countertops/domestic appliances	Lunch room	ICU-A	05 - May
150501772428.1.1	Bed railings from Patient Room A	bed railings	Patient room	ICU-B	05 - May
150501772429.1.1	Infusion pumps	medical device	Patient room	ICU-B	05 - May
150501772430.1.1	Screen monitors	medical device	Patient room	ICU-B	05 - May
150501772431.1.1	Curtain	wall/curtain	Patient room	ICU-B	05 - May

150501772432.1.1	Bed railings from Patient Room B	bed railings	Patient room	ICU-B	05 - May
150501772433.1.1	Utensils table	table	Patient room	ICU-B	05 - May
150501772434.1.1	Wall	wall/curtain	Patient room	ICU-B	05 - May
150501772435.1.1	Termometer	medical device	Patient room	ICU-B	05 - May
150501772436.1.1	Countertops, computer and phone	countertops/computer/phone	Nurse station	ICU-B	05 - May
150501772437.1.1	Refrigerator	equipment of common use	Nurse station	ICU-B	05 - May
150501772438.1.1	Computers	equipment of common use	Prescription room	ICU-B	05 - May
150501772439.1.1	Dispenser from the Hall	dispenser	Common place	ICU-B	05 - May
150501772440.1.1	Utensils from the Bath cart	medical device	Common place	ICU-B	05 - May
150501772441.1.1	Glicosimeter	medical device	Common place	ICU-B	05 - May
150501772442.1.1	Cufometer	medical device	Common place	ICU-B	05 - May
150501772443.1.1	Bath cart	medical device	Common place	ICU-B	05 - May
150501772444.1.1	Emergency cart	medical device	Common place	ICU-B	05 - May
150501772445.1.1	X-Raio cart	medical device	Common place	ICU-B	05 - May
150501772446.1.1	Ultrasound cart	medical device	Common place	ICU-B	05 - May
150501772447.1.1	Passant	medical device	Common place	ICU-B	05 - May
150501772448.1.1	Physiotherapeutic armchair	medical device	Common place	ICU-B	05 - May
150501772466.1.1	Bed railings from Patient Room A	bed railings	Patient room	ECU	05 - May
150501772467.1.1	Serum and gas support from Patient Room 1	serum/gas support	Patient room	ECU	05 - May
150501772468.1.1	Bed railings from Patient Room B	bed railings	Patient room	ECU	05 - May
150501772469.1.1	Serum and gas support from Patient Room 2	serum/gas support	Patient room	ECU	05 - May
150501772470.1.1	Bed railings from Patient Room C	bed railings	Patient room	ECU	05 - May
150501772471.1.1	Serum and gas support from Patient Room 3	serum/gas support	Patient room	ECU	05 - May
150501772472.1.1	Dispenser, water tap and flush from Patient Bathroom	dispenser/water tap/flush	Patient bathroom	ECU	05 - May
150501772473.1.1	Countertops, computer and phone	countertops/computer/phone	Nurse station	ECU	05 - May
150501772474.1.1	Dispenser and water tap	dispenser/water tap	Medicament room	ECU	05 - May
150501772475.1.1	Countertops and surfaces	countertops/surfaces	Medicament room	ECU	05 - May

150501772476.1.1	Chairs	chair	Medication room	ECU	05 - May
150501772477.1.1	Serum and gas support	serum/gas support	Medication room	ECU	05 - May
150501772478.1.1	Computers	equipment of common use	Prescription room	ECU	05 - May
150501772479.1.1	Sink	sink	Special procedures room	ECU	05 - May
150501772480.1.1	Bed	bed	Special procedures room	ECU	05 - May
150501772481.1.1	Countertops and surfaces	countertops/surfaces	Bandage room	ECU	05 - May
150501772482.1.1	Bed	bed	Clinic room	ECU	05 - May
150501772483.1.1	Computer table	table	Clinic room	ECU	05 - May
150501772484.1.1	Dispenser and water tap	dispenser/water tap	Clinic room	ECU	05 - May
150501772485.1.1	Chairs	chair	Reception	ECU	05 - May
150501772486.1.1	Table, countertops and domestic appliances	table/countertops/domestic appliances	Lunch room	ECU	05 - May
150501772487.1.1	Bed, table and chair	bed/table/chair	Resting room	ECU	05 - May
150501772488.1.1	Dispenser from the Hall	dispenser	Common place	ECU	05 - May
150501772489.1.1	Medication cart	medical device	Common place	ECU	05 - May
150603610414.1.1	Dispenser, water tap and flush from Patient Bathroom A	dispenser/water tap/flush	Patient bathroom	IU	06 - June
150603610415.1.1	Serum and gas support from Patient Room 1	serum/gas support	Patient room	IU	06 - June
150603610416.1.1	Bed railings from Patient Room A	bed railings	Patient room	IU	06 - June
150603610417.1.1	Dispenser, water tap and flush from Patient Bathroom B	dispenser/water tap/flush	Patient bathroom	IU	06 - June
150603610418.1.1	Bed railings from Patient Room B	bed railings	Patient room	IU	06 - June
150603610419.1.1	Serum and gas support from Patient Room 2	serum/gas support	Patient room	IU	06 - June
150603610420.1.1	Countertops, computer and phone	countertops/computer/phone	Nurse station	IU	06 - June
150603610421.1.1	Dispenser and water tap	dispenser/water tap	Medicament room	IU	06 - June
150603610422.1.1	Countertops and surfaces	countertops/surfaces	Medicament room	IU	06 - June
150603610423.1.1	Countertops and surfaces	countertops/surfaces	Bandage room	IU	06 - June
150603610424.1.1	Table, countertops and domestic appliances	table/countertops/domestic appliances	Lunch room	IU	06 - June
150603610425.1.1	Bed, table and chair	bed/table/chair	Resting room	IU	06 - June

150603610426.1.1	Dispenser from the Hall	dispenser	Common place	IU	06 - June
150603610427.1.1	Glicosimeter	medical device	Common place	IU	06 - June
150603610442.1.1	Dispenser, water tap and flush from Patient Bathroom A	dispenser/water tap/flush	Patient bathroom	MU	06 - June
150603610443.1.1	Bed railings from Patient Room B	bed railings	Patient Room	MU	06 - June
150603610444.1.1	Serum and gas support from Patient Room 1	serum/gas support	Patient room	MU	06 - June
150603610445.1.1	Dispenser, water tap and flush from Patient Bathroom B	dispenser/water tap/flush	Patient bathroom	MU	06 - June
150603610446.1.1	Bed railings from Patient Room A	bed railings	Patient room	MU	06 - June
150603610447.1.1	Serum and gas support from Patient Room 2	serum/gas support	Patient room	MU	06 - June
150603610448.1.1	Countertops, computer and phone	countertops/computer/phone	Nurse station	MU	06 - June
150603610449.1.1	Dispenser and water tap	dispenser/water tap	Medicament room	MU	06 - June
150603610450.1.1	Countertops and surfaces	countertops/surfaces	Medicament room	MU	06 - June
150603610451.1.1	Cabinets and chairs	cabinets/chair	Locker room	MU	06 - June
150603610452.1.1	Dispenser and water tap	dispenser/water tap	Locker room	MU	06 - June
150603610453.1.1	Table, countertops and domestic appliances	table/countertops/domestic appliances	Lunch room	MU	06 - June
150603610454.1.1	Bed, table and chair	bed/table/chair	Resting room	MU	06 - June
150603610455.1.1	Dispenser from the Hall	dispenser	Common place	MU	06 - June
150603610456.1.1	Glicosimeter	medical device	Common place	MU	06 - June
150603610465.1.1	Bed railings	bed railings	Recovery room	SC	06 - June
150603610466.1.1	Serum and gas support	serum/gas support	Recovery room	SC	06 - June
150603610467.1.1	Countertops and surfaces	countertops/surfaces	Recovery room	SC	06 - June
150603610468.1.1	Surgical table from Room 1	bed	Surgery room	SC	06 - June
150603610469.1.1	Oxigen buttons	surgery device	Surgery room	SC	06 - June
150603610470.1.1	Laryngoscope instrument tray	surgery device	Surgery room	SC	06 - June
150603610471.1.1	Anesthesia syringes	surgery device	Surgery room	SC	06 - June
150603610472.1.1	Surgical table from Room 2	bed	Surgery room	SC	06 - June
150603610473.1.1	Surgical light	surgery device	Surgery room	SC	06 - June
150603610474.1.1	Syringes	surgery device	Surgery room	SC	06 - June

150603610475.1.1	Electric scalpel	surgery device	Surgery room	SC	06 - June
150603610476.1.1	Cabinets and chairs	cabinets/chair	Locker room	SC	06 - June
150603610477.1.1	Dispenser and water tap	dispenser/water tap	Locker room	SC	06 - June
150603610478.1.1	Bed, table and chair	bed/table/chair	Resting room	SC	06 - June
150603610479.1.1	Table, countertops and domestic appliances	table/countertops/domestic appliances	Lunch room	SC	06 - June
150603610480.1.1	Computer	equipment of common use	Nurse chief room	SC	06 - June
150603610481.1.1	Stretcher from the Hall	bed	Common place	SC	06 - June
150603610482.1.1	Dispenser from the Hall	dispenser	Common place	SC	06 - June
150603610497.1.1	Bed railings from Patient Room A	bed railings	Patient room	ICU-A	06 - June
150603610498.1.1	Infusion pumps	medical device	Patient room	ICU-A	06 - June
150603610499.1.1	Screen monitors	medical device	Patient room	ICU-A	06 - June
150603610500.1.1	Curtain	wall/curtain	Patient room	ICU-A	06 - June
150603610501.1.1	Bed railings from Patient Room B	bed railings	Patient room	ICU-A	06 - June
150603610502.1.1	Utensils table	table	Patient room	ICU-A	06 - June
150603610503.1.1	Wall	wall/curtain	Patient room	ICU-A	06 - June
150603610504.1.1	Termometer	medical device	Patient room	ICU-A	06 - June
150603610505.1.1	Countertops, computer and phone	countertops/computer/phone	Nurse station	ICU-A	06 - June
150603610506.1.1	Clipboards	equipment of common use	Nurse station	ICU-A	06 - June
150603610507.1.1	Computers	equipment of common use	Prescription room	ICU-A	06 - June
150603610508.1.1	SEquipMateriais - EquipLimpos	medical device	Medical device room	ICU-A	06 - June
150603610509.1.1	Cabinets and chairs	cabinets/chair	Locker room	ICU-A	06 - June
150603610510.1.1	Dispenser, water tap and flush from Bathroom	dispenser/water tap/flush	Locker room	ICU-A	06 - June
150603610511.1.1	Countertops and surfaces	countertops/surfaces	Purge room	ICU-A	06 - June
150603610512.1.1	Dryer	equipment of common use	Purge room	ICU-A	06 - June
150603610513.1.1	Bed, table and chair	bed/table/chair	Resting room	ICU-A	06 - June
150603610514.1.1	Bed, table and chair	bed/table/chair	Resting room	ICU-A	06 - June
150603610515.1.1	Table, countertops and domestic appliances	table/countertops/domestic appliances	Lunch room	ICU-A	06 - June
150603610528.1.1	Bed railings from Patient Room A	bed railings	Patient room	ICU-B	06 - June

150603610529.1.1	Infusion pumps	medical device	Patient room	ICU-B	06 - June
150603610530.1.1	Screen monitors	medical device	Patient room	ICU-B	06 - June
150603610531.1.1	Curtain	wall/curtain	Patient room	ICU-B	06 - June
150603610532.1.1	Bed railings from Patient Room B	bed railings	Patient room	ICU-B	06 - June
150603610533.1.1	Utensils table	table	Patient room	ICU-B	06 - June
150603610534.1.1	Wall	wall/curtain	Patient room	ICU-B	06 - June
150603610535.1.1	Termometer	medical device	Patient room	ICU-B	06 - June
150603610536.1.1	Countertops, computer and phone	countertops/computer/phone	Nurse station	ICU-B	06 - June
150603610537.1.1	Refrigerator	equipment of common use	Nurse station	ICU-B	06 - June
150603610538.1.1	Computers	equipment of common use	Prescription room	ICU-B	06 - June
150603610539.1.1	Dispenser from the Hall	dispenser	Common place	ICU-B	06 - June
150603610540.1.1	Utensils from the Bath cart	medical device	Common place	ICU-B	06 - June
150603610541.1.1	Glicosimeter	medical device	Common place	ICU-B	06 - June
150603610542.1.1	Cufometer	medical device	Common place	ICU-B	06 - June
150603610543.1.1	Bath cart	medical device	Common place	ICU-B	06 - June
150603610544.1.1	Emergency cart	medical device	Common place	ICU-B	06 - June
150603610545.1.1	X-Raio cart	medical device	Common place	ICU-B	06 - June
150603610546.1.1	Ultrasound cart	medical device	Common place	ICU-B	06 - June
150603610547.1.1	Passant	medical device	Common place	ICU-B	06 - June
150603610548.1.1	Physiotherapeutic armchair	medical device	Common place	ICU-B	06 - June
150603610566.1.1	Bed railings from Patient Room A	bed railings	Patient room	ECU	06 - June
150603610567.1.1	Serum and gas support from Patient Room 1	serum/gas support	Patient room	ECU	06 - June
150603610568.1.1	Bed railings from Patient Room B	bed railings	Patient room	ECU	06 - June
150603610569.1.1	Serum and gas support from Patient Room 2	serum/gas support	Patient room	ECU	06 - June
150603610570.1.1	Bed railings from Patient Room C	bed railings	Patient room	ECU	06 - June
150603610571.1.1	Serum and gas support from Patient Room 3	serum/gas support	Patient room	ECU	06 - June
150603610572.1.1	Dispenser, water tap and flush from Patient Bathroom	dispenser/water tap/flush	Patient bathroom	ECU	06 - June

150603610573.1.1	Countertops, computer and phone	countertops/computer/phone	Nurse station	ECU	06 - June
150603610574.1.1	Dispenser and water tap	dispenser/water tap	Medicament room	ECU	06 - June
150603610575.1.1	Countertops and surfaces	countertops/surfaces	Medicament room	ECU	06 - June
150603610576.1.1	Chairs	chair	Medication room	ECU	06 - June
150603610577.1.1	Serum and gas support	serum/gas support	Medication room	ECU	06 - June
150603610578.1.1	Computers	equipment of common use	Prescription room	ECU	06 - June
150603610579.1.1	Sink	sink	Special procedures room	ECU	06 - June
150603610580.1.1	Bed	bed	Special procedures room	ECU	06 - June
150603610581.1.1	Countertops and surfaces	countertops/surfaces	Bandage room	ECU	06 - June
150603610582.1.1	Bed	bed	Clinic room	ECU	06 - June
150603610583.1.1	Computer table	table	Clinic room	ECU	06 - June
150603610584.1.1	Dispenser and water tap	dispenser/water tap	Clinic room	ECU	06 - June
150603610585.1.1	Chairs	chair	Reception	ECU	06 - June
150603610586.1.1	Table, countertops and domestic appliances	table/countertops/domestic appliances	Lunch room	ECU	06 - June
150603610587.1.1	Bed, table and chair	bed/table/chair	Resting room	ECU	06 - June
150701108514.1.1	Dispenser, water tap and flush from Patient Bathroom A	dispenser/water tap/flush	Patient bathroom	IU	07 - July
150701108515.1.1	Serum and gas support from Patient Room 1	serum/gas support	Patient room	IU	07 - July
150701108516.1.1	Bed railings from Patient Room A	bed railings	Patient room	IU	07 - July
150701108517.1.1	Dispenser, water tap and flush from Patient Bathroom B	dispenser/water tap/flush	Patient bathroom	IU	07 - July
150701108518.1.1	Bed railings from Patient Room B	bed railings	Patient room	IU	07 - July
150701108519.1.1	Serum and gas support from Patient Room 2	serum/gas support	Patient room	IU	07 - July
150701108520.1.1	Countertops, computer and phone	countertops/computer/phone	Nurse station	IU	07 - July
150701108521.1.1	Dispenser and water tap	dispenser/water tap	Medicament room	IU	07 - July
150701108522.1.1	Countertops and surfaces	countertops/surfaces	Medicament room	IU	07 - July
150701108523.1.1	Countertops and surfaces	countertops/surfaces	Bandage room	IU	07 - July
150701108524.1.1	Table, countertops and domestic appliances	table/countertops/domestic appliances	Lunch room	IU	07 - July

150701108525.1.1	Bed, table and chair	bed/table/chair	Resting room	IU	07 - July
150701108526.1.1	Dispenser from the Hall	dispenser	Common place	IU	07 - July
150701108527.1.1	Glicosimeter	medical device	Common place	IU	07 - July
150701108542.1.1	Dispenser, water tap and flush from Patient Bathroom A	dispenser/water tap/flush	Patient bathroom	MU	07 - July
150701108543.1.1	Bed railings from Patient Room A	bed railings	Patient room	MU	07 - July
150701108544.1.1	Serum and gas support from Patient Room 1	serum/gas support	Patient room	MU	07 - July
150701108545.1.1	Dispenser, water tap and flush from Patient Bathroom B	dispenser/water tap/flush	Patient bathroom	MU	07 - July
150701108546.1.1	Bed railings from Patient Room B	bed railings	Patient room	MU	07 - July
150701108547.1.1	Serum and gas support from Patient Room 2	serum/gas support	Patient room	MU	07 - July
150701108548.1.1	Countertops, computer and phone	countertops/computer/phone	Nurse station	MU	07 - July
150701108549.1.1	Dispenser and water tap	dispenser/water tap	Medicament room	MU	07 - July
150701108550.1.1	Countertops and surfaces	countertops/surfaces	Medicament room	MU	07 - July
150701108551.1.1	Cabinets and chairs	cabinets/chair	Locker room	MU	07 - July
150701108552.1.1	Dispenser and water tap	dispenser/water tap	Locker room	MU	07 - July
150701108553.1.1	Table, countertops and domestic appliances	table/countertops/domestic appliances	Lunch room	MU	07 - July
150701108554.1.1	Bed, table and chair	bed/table/chair	Resting room	MU	07 - July
150701108555.1.1	Dispenser from the Hall	dispenser	Common place	MU	07 - July
150701108556.1.1	Glicosimeter	medical device	Common place	MU	07 - July
150701108565.1.1	Bed railings	bed railings	Recovery room	SC	07 - July
150701108566.1.1	Serum and gas support	serum/gas support	Recovery room	SC	07 - July
150701108567.1.1	Countertops and surfaces	countertops/surfaces	Recovery room	SC	07 - July
150701108568.1.1	Surgical table from Room 1	bed	Surgery room	SC	07 - July
150701108569.1.1	Oxigen buttons	surgery device	Surgery room	SC	07 - July
150701108570.1.1	Laryngoscope instrument tray	surgery device	Surgery room	SC	07 - July
150701108571.1.1	Anesthesia syringes	surgery device	Surgery room	SC	07 - July
150701108572.1.1	Surgical table from Room 2	bed	Surgery room	SC	07 - July
150701108573.1.1	Surgical light	surgery device	Surgery room	SC	07 - July

150701108574.1.1	Syringes	surgery device	Surgery room	SC	07 - July
150701108575.1.1	Electric scalpel	surgery device	Surgery room	SC	07 - July
150701108576.1.1	Cabinets and chairs	cabinets/chair	Locker room	SC	07 - July
150701108577.1.1	Dispenser and water tap	dispenser/water tap	Locker room	SC	07 - July
150701108578.1.1	Bed, table and chair	bed/table/chair	Resting room	SC	07 - July
150701108579.1.1	Table, countertops and domestic appliances	table/countertops/domestic appliances	Lunch room	SC	07 - July
150701108580.1.1	Computer	equipment of common use	Nurse chief room	SC	07 - July
150701108581.1.1	Stretcher from the Hall	bed	Common place	SC	07 - July
150701108582.1.1	Dispenser from the Hall	dispenser	Common place	SC	07 - July
150701108597.1.1	Bed railings from Patient Room A	bed railings	Patient room	ICU-A	07 - July
150701108598.1.1	Infusion pumps	medical device	Patient room	ICU-A	07 - July
150701108599.1.1	Screen monitors	medical device	Patient room	ICU-A	07 - July
150701108600.1.1	Curtain	wall/curtain	Patient room	ICU-A	07 - July
150701108601.1.1	Bed railings from Patient Room B	bed railings	Patient room	ICU-A	07 - July
150701108602.1.1	Utensils table	table	Patient room	ICU-A	07 - July
150701108603.1.1	Wall	wall/curtain	Patient room	ICU-A	07 - July
150701108604.1.1	Termometer	medical device	Patient room	ICU-A	07 - July
150701108605.1.1	Countertops, computer and phone	countertops/computer/phone	Nurse station	ICU-A	07 - July
150701108606.1.1	Clipboards	equipment of common use	Nurse station	ICU-A	07 - July
150701108607.1.1	Computers	equipment of common use	Prescription room	ICU-A	07 - July
150701108608.1.1	SEquipMateriais-EquipLimpos	medical device	Medical device room	ICU-A	07 - July
150701108609.1.1	Cabinets and chairs	cabinets/chair	Locker room	ICU-A	07 - July
150701108610.1.1	Dispenser, water tap and flush from Bathroom	dispenser/water tap/flush	Locker room	ICU-A	07 - July
150701108611.1.1	Countertops and surfaces	countertops/surfaces	Purge room	ICU-A	07 - July
150701108612.1.1	Dryer	equipment of common use	Purge room	ICU-A	07 - July
150701108613.1.1	Bed, table and chair	bed/table/chair	Resting room	ICU-A	07 - July
150701108614.1.1	Bed, table and chair	bed/table/chair	Resting room	ICU-A	07 - July
150701108615.1.1	Table, countertops and domestic appliances	table/countertops/domestic appliances	Lunch room	ICU-A	07 - July

150701108628.1.1	Bed railings from Patient Room A	bed railings	Patient room	ICU-B	07 - July
150701108629.1.1	Infusion pumps	medical device	Patient room	ICU-B	07 - July
150701108630.1.1	Screen monitors	medical device	Patient room	ICU-B	07 - July
150701108631.1.1	Curtain	wall/curtain	Patient room	ICU-B	07 - July
150701108632.1.1	Bed railings from Patient Room B	bed railings	Patient room	ICU-B	07 - July
150701108633.1.1	Utensils table	table	Patient room	ICU-B	07 - July
150701108634.1.1	Wall	wall/curtain	Patient room	ICU-B	07 - July
150701108635.1.1	Termometer	medical device	Patient room	ICU-B	07 - July
150701108636.1.1	Countertops, computer and phone	countertops/computer/phone	Nurse station	ICU-B	07 - July
150701108637.1.1	Refrigerator	equipment of common use	Nurse station	ICU-B	07 - July
150701108638.1.1	Computers	equipment of common use	Prescription room	ICU-B	07 - July
150701108639.1.1	Dispenser from the Hall	dispenser	Common place	ICU-B	07 - July
150701108640.1.1	Utensils from the Bath cart	medical device	Common place	ICU-B	07 - July
150701108641.1.1	Glicosimeter	medical device	Common place	ICU-B	07 - July
150701108642.1.1	Cufometer	medical device	Common place	ICU-B	07 - July
150701108643.1.1	Bath cart	medical device	Common place	ICU-B	07 - July
150701108644.1.1	Emergency cart	medical device	Common place	ICU-B	07 - July
150701108645.1.1	X-Raio cart	medical device	Common place	ICU-B	07 - July
150701108646.1.1	Ultrasound cart	medical device	Common place	ICU-B	07 - July
150701108647.1.1	Passant	medical device	Common place	ICU-B	07 - July
150701108648.1.1	Physiotherapeutic armchair	medical device	Common place	ICU-B	07 - July
150701108666.1.1	Bed railings from Patient Room A	bed railings	Patient room	ECU	07 - July
150701108667.1.1	Serum and gas support from Patient Room 1	serum/gas support	Patient room	ECU	07 - July
150701108668.1.1	Bed railings from Patient Room B	bed railings	Patient room	ECU	07 - July
150701108669.1.1	Serum and gas support from Patient Room 2	serum/gas support	Patient room	ECU	07 - July
150701108670.1.1	Bed railings from Patient Room C	bed railings	Patient room	ECU	07 - July
150701108671.1.1	Serum and gas support from Patient Room 3	serum/gas support	Patient room	ECU	07 - July

150701108672.1.1	Dispenser, water tap and flush from Patient Bathroom	dispenser/water tap/flush	Patient bathroom	ECU	07 - July
150701108673.1.1	Countertops, computer and phone	countertops/computer/phone	Nurse station	ECU	07 - July
150701108674.1.1	Dispenser and water tap	dispenser/water tap	Medicament room	ECU	07 - July
150701108675.1.1	Countertops and surfaces	countertops/surfaces	Medicament room	ECU	07 - July
150701108676.1.1	Chairs	chair	Medication room	ECU	07 - July
150701108677.1.1	Serum and gas support	serum/gas support	Medication room	ECU	07 - July
150701108678.1.1	Computers	equipment of common use	Prescription room	ECU	07 - July
150701108679.1.1	Sink	sink	Special procedures room	ECU	07 - July
150701108680.1.1	Bed	bed	Special procedures room	ECU	07 - July
150701108681.1.1	Countertops and surfaces	countertops/surfaces	Bandage room	ECU	07 - July
150701108682.1.1	Bed	bed	Clinic room	ECU	07 - July
150701108683.1.1	Computer table	table	Clinic room	ECU	07 - July
150701108684.1.1	Dispenser and water tap	dispenser/water tap	Clinic room	ECU	07 - July
150701108685.1.1	Chairs	chair	Reception	ECU	07 - July
150701108686.1.1	Table, countertops and domestic appliances	table/countertops/domestic appliances	Lunch room	ECU	07 - July
150701108687.1.1	Bed, table and chair	bed/table/chair	Resting room	ECU	07 - July
150701108688.1.1	Dispenser from the Hall	dispenser	Common place	ECU	07 - July
150701108689.1.1	Medication cart	medical device	Common place	ECU	07 - July
150804524314.2.1	Dispenser, water tap and flush from Patient Bathroom A	dispenser/water tap/flush	Patient bathroom	IU	08 - August
150804524315.2.1	Serum and gas support from Patient Room 1	serum/gas support	Patient room	IU	08 - August
150804524316.2.1	Bed railings from Patient Room A	bed railings	Patient room	IU	08 - August
150804524317.2.1	Dispenser, water tap and flush from Patient Bathroom B	dispenser/water tap/flush	Patient bathroom	IU	08 - August
150804524318.2.1	Bed railings from Patient Room B	bed railings	Patient room	IU	08 - August
150804524319.2.1	Serum and gas support from Patient Room 2	serum/gas support	Patient room	IU	08 - August
150804524320.2.1	Countertops, computer and phone	countertops/computer/phone	Nurse station	IU	08 - August
150804524321.2.1	Dispenser and water tap	dispenser/water tap	Medicament room	IU	08 - August

150804524322.2.1	Countertops and surfaces	countertops/surfaces	Medicament room	IU	08 - August
150804524323.2.1	Countertops and surfaces	countertops/surfaces	Bandage room	IU	08 - August
150804524324.2.1	Table, countertops and domestic appliances	table/countertops/domestic appliances	Lunch room	IU	08 - August
150804524325.2.1	Bed, table and chair	bed/table/chair	Resting room	IU	08 - August
150804524326.2.1	Dispenser from the Hall	dispenser	Common place	IU	08 - August
150804524327.2.1	Glicosimeter	medical device	Common place	IU	08 - August
150804524342.2.1	Dispenser, water tap and flush from Patient Bathroom A	dispenser/water tap/flush	Patient bathroom	MU	08 - August
150804524343.2.1	Bed railings from Patient Room A	bed railings	Patient room	MU	08 - August
150804524344.2.1	Serum and gas support from Patient Room 1	serum/gas support	Patient room	MU	08 - August
150804524345.2.1	Dispenser, water tap and flush from Patient Bathroom B	dispenser/water tap/flush	Patient bathroom	MU	08 - August
150804524346.2.1	Bed railings from Patient Room B	bed railings	Patient room	MU	08 - August
150804524347.2.1	Serum and gas support from Patient Room 2	serum/gas support	Patient room	MU	08 - August
150804524348.2.1	Countertops, computer and phone	countertops/computer/phone	Nurse station	MU	08 - August
150804524349.2.1	Dispenser and water tap	dispenser/water tap	Medicament room	MU	08 - August
150804524350.2.1	Countertops and surfaces	countertops/surfaces	Medicament room	MU	08 - August
150804524351.2.1	Cabinets and chairs	cabinets/chair	Locker room	MU	08 - August
150804524352.2.1	Dispenser and water tap	dispenser/water tap	Locker room	MU	08 - August
150804524353.2.1	Table, countertops and domestic appliances	table/countertops/domestic appliances	Lunch room	MU	08 - August
150804524354.2.1	Bed, table and chair	bed/table/chair	Resting room	MU	08 - August
150804524355.2.1	Dispenser from the Hall	dispenser	Common place	MU	08 - August
150804524356.2.1	Glicosimeter	medical device	Common place	MU	08 - August
150804524365.2.1	Bed railings	bed railings	Recovery room	SC	08 - August
150804524366.2.1	Serum and gas support	serum/gas support	Recovery room	SC	08 - August
150804524367.2.1	Countertops and surfaces	countertops/surfaces	Recovery room	SC	08 - August
150804524368.2.1	Surgical table from Room 1	bed	Surgery room	SC	08 - August
150804524369.2.1	Oxigen buttons	surgery device	Surgery room	SC	08 - August

150804524370.2.1	Laryngoscope instrument tray	surgery device	Surgery room	SC	08 - August
150804524371.2.1	Anesthesia syringes	surgery device	Surgery room	SC	08 - August
150804524372.2.1	Surgical table from Room 2	bed	Surgery room	SC	08 - August
150804524373.2.1	Surgical light	surgery device	Surgery room	SC	08 - August
150804524374.2.1	Syringes	surgery device	Surgery room	SC	08 - August
150804524375.2.1	Electric scalpel	surgery device	Surgery room	SC	08 - August
150804524376.2.1	Cabinets and chairs	cabinets/chair	Locker room	SC	08 - August
150804524377.2.1	Dispenser and water tap	dispenser/water tap	Locker room	SC	08 - August
150804524378.2.1	Bed, table and chair	bed/table/chair	Resting room	SC	08 - August
150804524379.2.1	Table, countertops and domestic appliances	table/countertops/domestic appliances	Lunch room	SC	08 - August
150804524380.2.1	Computer	equipment of common use	Nurse chief room	SC	08 - August
150804524381.2.1	Stretcher from the Hall	bed	Common place	SC	08 - August
150804524382.2.1	Dispenser from the Hall	dispenser	Common place	SC	08 - August
150804524397.2.1	Bed railings from Patient Room A	bed railings	Patient room	ICU-A	08 - August
150804524398.2.1	Infusion pumps	medical device	Patient room	ICU-A	08 - August
150804524399.2.1	Screen monitors	medical device	Patient room	ICU-A	08 - August
150804524400.2.1	Curtain	wall/curtain	Patient room	ICU-A	08 - August
150804524401.2.1	Bed railings from Patient Room B	bed railings	Patient room	ICU-A	08 - August
150804524402.2.1	Utensils table	table	Patient room	ICU-A	08 - August
150804524403.2.1	Wall	wall/curtain	Patient room	ICU-A	08 - August
150804524404.2.1	Termometer	medical device	Patient room	ICU-A	08 - August
150804524405.2.1	Countertops, computer and phone	countertops/computer/phone	Nurse station	ICU-A	08 - August
150804524406.2.1	Clipboards	equipment of common use	Nurse station	ICU-A	08 - August
150804524407.2.1	Computers	equipment of common use	Prescription room	ICU-A	08 - August
150804524408.2.1	SEquipMateriais-EquipLimpos	medical device	Medical device room	ICU-A	08 - August
150804524409.2.1	Cabinets and chairs	cabinets/chair	Locker room	ICU-A	08 - August
150804524410.2.1	Dispenser, water tap and flush from Bathroom	dispenser/water tap/flush	Locker room	ICU-A	08 - August
150804524411.2.1	Countertops and surfaces	countertops/surfaces	Purge room	ICU-A	08 - August

150804524412.2.1	Dryer	equipment of common use	Purge room	ICU-A	08 - August
150804524413.2.1	Bed, table and chair	bed/table/chair	Resting room	ICU-A	08 - August
150804524414.2.1	Bed, table and chair	bed/table/chair	Resting room	ICU-A	08 - August
150804524415.2.1	Table, countertops and domestic appliances	table/countertops/domestic appliances	Lunch room	ICU-A	08 - August
150804524428.2.1	Bed railings from Patient Room A	bed railings	Patient room	ICU-B	08 - August
150804524429.2.1	Infusion pumps	medical device	Patient room	ICU-B	08 - August
150804524430.2.1	Screen monitors	medical device	Patient room	ICU-B	08 - August
150804524431.2.1	Curtain	wall/curtain	Patient room	ICU-B	08 - August
150804524432.2.1	Bed railings from Patient Room B	bed railings	Patient room	ICU-B	08 - August
150804524433.2.1	Utensils table	table	Patient room	ICU-B	08 - August
150804524434.2.1	Wall	wall/curtain	Patient room	ICU-B	08 - August
150804524435.2.1	Termometer	medical device	Patient room	ICU-B	08 - August
150804524436.2.1	Countertops, computer and phone	countertops/computer/phone	Nurse station	ICU-B	08 - August
150804524437.2.1	Refrigerator	equipment of common use	Nurse station	ICU-B	08 - August
150804524438.2.1	Computers	equipment of common use	Prescription room	ICU-B	08 - August
150804524439.2.1	Dispenser from the Hall	dispenser	Common place	ICU-B	08 - August
150804524440.2.1	Utensils from the Bath cart	medical device	Common place	ICU-B	08 - August
150804524441.2.1	Glicosimeter	medical device	Common place	ICU-B	08 - August
150804524442.2.1	Cufometer	medical device	Common place	ICU-B	08 - August
150804524443.2.1	Bath cart	medical device	Common place	ICU-B	08 - August
150804524444.2.1	Emergency cart	medical device	Common place	ICU-B	08 - August
150804524445.2.1	X-Raio cart	medical device	Common place	ICU-B	08 - August
150804524446.2.1	Ultrasound cart	medical device	Common place	ICU-B	08 - August
150804524447.2.1	Passant	medical device	Common place	ICU-B	08 - August
150804524448.2.1	Physiotherapeutic armchair	medical device	Common place	ICU-B	08 - August
150804524466.2.1	Bed railings from Patient Room A	bed railings	Patient room	ECU	08 - August
150804524467.2.1	Serum and gas support from Patient Room 1	serum/gas support	Patient room	ECU	08 - August
150804524468.2.1	Bed railings from Patient Room B	bed railings	Patient room	ECU	08 - August

150804524469.2.1	Serum and gas support from Patient Room 2	serum/gas support	Patient room	ECU	08 - August
150804524470.2.1	Bed railings from Patient Room C	bed railings	Patient room	ECU	08 - August
150804524471.2.1	Serum and gas support from Patient Room 3	serum/gas support	Patient room	ECU	08 - August
150804524472.2.1	Dispenser, water tap and flush from Patient Bathroom	dispenser/water tap/flush	Patient bathroom	ECU	08 - August
150804524473.2.1	Countertops, computer and phone	countertops/computer/phone	Nurse station	ECU	08 - August
150804524474.2.1	Dispenser and water tap	dispenser/water tap	Medicament room	ECU	08 - August
150804524475.2.1	Countertops and surfaces	countertops/surfaces	Medicament room	ECU	08 - August
150804524476.2.1	Chairs	chair	Medication room	ECU	08 - August
150804524477.2.1	Serum and gas support	serum/gas support	Medication room	ECU	08 - August
150804524478.2.1	Computers	equipment of common use	Prescription room	ECU	08 - August
150804524479.2.1	Sink	sink	Special procedures room	ECU	08 - August
150804524480.2.1	Bed	bed	Special procedures room	ECU	08 - August
150804524481.2.1	Countertops and surfaces	countertops/surfaces	Bandage room	ECU	08 - August
150804524482.2.1	Bed	bed	Clinic room	ECU	08 - August
150804524483.2.1	Computer table	table	Clinic room	ECU	08 - August
150804524484.2.1	Dispenser and water tap	dispenser/water tap	Clinic room	ECU	08 - August
150804524485.2.1	Chairs	chair	Reception	ECU	08 - August
150804524486.2.1	Table, countertops and domestic appliances	table/countertops/domestic appliances	Lunch room	ECU	08 - August
150804524487.2.1	Bed, table and chair	bed/table/chair	Resting room	ECU	08 - August
150804524488.2.1	Dispenser from the Hall	dispenser	Common place	ECU	08 - August
150804524489.2.1	Medication cart	medical device	Common place	ECU	08 - August
150826658414.1.1	Dispenser, water tap and flush from Patient Bathroom B	dispenser/water tap/flush	Patient bathroom	IU	09 - September
150826658415.1.1	Serum and gas support from Patient Room 1	serum/gas support	Patient room	IU	09 - September
150826658416.1.1	Bed railings from Patient Room A	bed railings	Patient room	IU	09 - September
150826658418.1.1	Bed railings from Patient Room B	bed railings	Patient room	IU	09 - September

150826658419.1.1	Serum and gas support from Patient Room 2	serum/gas support	Patient room	IU	09 - September
150826658420.1.1	Countertops, computer and phone	countertops/computer/phone	Nurse station	IU	09 - September
150826658421.1.1	Dispenser and water tap	dispenser/water tap	Medicament room	IU	09 - September
150826658422.1.1	Countertops and surfaces	countertops/surfaces	Medicament room	IU	09 - September
150826658423.1.1	Countertops and surfaces	countertops/surfaces	Bandage room	IU	09 - September
150826658424.1.1	Table, countertops and domestic appliances	table/countertops/domestic appliances	Lunch room	IU	09 - September
150826658425.1.1	Bed, table and chair	bed/table/chair	Resting room	IU	09 - September
150826658426.1.1	Dispenser from the Hall	dispenser	Common place	IU	09 - September
150826658427.1.1	Glicosimeter	medical device	Common place	IU	09 - September
150826658442.1.1	Dispenser, water tap and flush from Patient Bathroom A	dispenser/water tap/flush	Patient bathroom	MU	09 - September
150826658443.1.1	Bed railings from Patient Room A	bed railings	Patient room	MU	09 - September
150826658444.1.1	Serum and gas support from Patient Room 1	serum/gas support	Patient room	MU	09 - September
150826658445.1.1	Dispenser, water tap and flush from Patient Bathroom B	dispenser/water tap/flush	Patient bathroom	MU	09 - September
150826658446.1.1	Bed railings from Patient Room B	bed railings	Patient room	MU	09 - September
150826658447.1.1	Serum and gas support from Patient Room 2	serum/gas support	Patient room	MU	09 - September
150826658448.1.1	Countertops, computer and phone	countertops/computer/phone	Nurse station	MU	09 - September
150826658449.1.1	Dispenser and water tap	dispenser/water tap	Medicament room	MU	09 - September
150826658450.1.1	Countertops and surfaces	countertops/surfaces	Medicament room	MU	09 - September
150826658451.1.1	Cabinets and chairs	cabinets/chair	Locker room	MU	09 - September
150826658452.1.1	Dispenser and water tap	dispenser/water tap	Locker room	MU	09 - September
150826658453.1.1	Table, countertops and domestic appliances	table/countertops/domestic appliances	Lunch room	MU	09 - September
150826658454.1.1	Bed, table and chair	bed/table/chair	Resting room	MU	09 - September
150826658455.1.1	Dispenser from the Hall	dispenser	Common place	MU	09 - September
150826658456.1.1	Glicosimeter	medical device	Common place	MU	09 - September
150826658465.1.1	Bed railings	bed railings	Recovery room	SC	09 - September

150826658466.1.1	Serum and gas support	serum/gas support	Recovery room	SC	09 - September
150826658467.1.1	Countertops and surfaces	countertops/surfaces	Recovery room	SC	09 - September
150826658468.1.1	Surgical table from Room 1	bed	Surgery room	SC	09 - September
150826658469.1.1	Oxigen buttons	surgery device	Surgery room	SC	09 - September
150826658470.1.1	Laryngoscope instrument tray	surgery device	Surgery room	SC	09 - September
150826658471.1.1	Anesthesia syringes	surgery device	Surgery room	SC	09 - September
150826658472.1.1	Surgical table from Room 2	bed	Surgery room	SC	09 - September
150826658473.1.1	Surgical light	surgery device	Surgery room	SC	09 - September
150826658474.1.1	Syringes	surgery device	Surgery room	SC	09 - September
150826658475.1.1	Electric scalpel	surgery device	Surgery room	SC	09 - September
150826658476.1.1	Cabinets and chairs	cabinets/chair	Locker room	SC	09 - September
150826658477.1.1	Dispenser and water tap	dispenser/water tap	Locker room	SC	09 - September
150826658478.1.1	Bed, table and chair	bed/table/chair	Resting room	SC	09 - September
150826658479.1.1	Table, countertops and domestic appliances	table/countertops/domestic appliances	Lunch room	SC	09 - September
150826658480.1.1	Computer	equipment of common use	Nurse chief room	SC	09 - September
150826658481.1.1	Stretcher from the Hall	bed	Common place	SC	09 - September
150826658482.1.1	Dispenser from the Hall	dispenser	Common place	SC	09 - September
150826658497.1.1	Bed railings from Patient Room A	bed railings	Patient room	ICU-A	09 - September
150826658498.1.1	Infusion pumps	medical device	Patient room	ICU-A	09 - September
150826658499.1.1	Screen monitors	medical device	Patient room	ICU-A	09 - September
150826658500.1.1	Curtain	wall/curtain	Patient room	ICU-A	09 - September
150826658501.1.1	Bed railings from Patient Room B	bed railings	Patient room	ICU-A	09 - September
150826658502.1.1	Utensils table	table	Patient room	ICU-A	09 - September
150826658503.1.1	Wall	wall/curtain	Patient room	ICU-A	09 - September
150826658504.1.1	Termometer	medical device	Patient room	ICU-A	09 - September
150826658505.1.1	Countertops, computer and phone	countertops/computer/phone	Nurse station	ICU-A	09 - September
150826658506.1.1	Clipboards	equipment of common use	Nurse station	ICU-A	09 - September
150826658507.1.1	Computers	equipment of common use	Prescription room	ICU-A	09 - September
150826658508.1.1	SEquipMateriais-EquipLimpos	medical device	Medical device room	ICU-A	09 - September

150826658509.1.1	Cabinets and chairs	cabinets/chair	Locker room	ICU-A	09 - September
150826658510.1.1	Dispenser, water tap and flush from Bathroom	dispenser/water tap/flush	Locker room	ICU-A	09 - September
150826658511.1.1	Countertops and surfaces	countertops/surfaces	Purge room	ICU-A	09 - September
150826658512.1.1	Dryer	equipment of common use	Purge room	ICU-A	09 - September
150826658513.1.1	Bed, table and chair	bed/table/chair	Resting room	ICU-A	09 - September
150826658514.1.1	Bed, table and chair	bed/table/chair	Resting room	ICU-A	09 - September
150826658515.1.1	Table, countertops and domestic appliances	table/countertops/domestic appliances	Lunch room	ICU-A	09 - September
150826658528.1.1	Bed railings from Patient Room A	bed railings	Patient room	ICU-B	09 - September
150826658529.1.1	Infusion pumps	medical device	Patient room	ICU-B	09 - September
150826658530.1.1	Screen monitors	medical device	Patient room	ICU-B	09 - September
150826658531.1.1	Curtain	wall/curtain	Patient room	ICU-B	09 - September
150826658532.1.1	Bed railings from Patient Room B	bed railings	Patient room	ICU-B	09 - September
150826658533.1.1	Utensils table	table	Patient room	ICU-B	09 - September
150826658534.1.1	Wall	wall/curtain	Patient room	ICU-B	09 - September
150826658535.1.1	Termometer	medical device	Patient room	ICU-B	09 - September
150826658536.1.1	Countertops, computer and phone	countertops/computer/phone	Nurse station	ICU-B	09 - September
150826658537.1.1	Refrigerator	equipment of common use	Nurse station	ICU-B	09 - September
150826658538.1.1	Computers	equipment of common use	Prescription room	ICU-B	09 - September
150826658539.1.1	Dispenser from the Hall	dispenser	Common place	ICU-B	09 - September
150826658540.1.1	Utensils from the Bath cart	medical device	Common place	ICU-B	09 - September
150826658541.1.1	Glicosimeter	medical device	Common place	ICU-B	09 - September
150826658542.1.1	Cufometer	medical device	Common place	ICU-B	09 - September
150826658543.1.1	Bath cart	medical device	Common place	ICU-B	09 - September
150826658544.1.1	Emergency cart	medical device	Common place	ICU-B	09 - September
150826658545.1.1	X-Raio cart	medical device	Common place	ICU-B	09 - September
150826658546.1.1	Ultrasound cart	medical device	Common place	ICU-B	09 - September
150826658547.1.1	Passant	medical device	Common place	ICU-B	09 - September
150826658548.1.1	Physiotherapeutic armchair	medical device	Common place	ICU-B	09 - September

150826658566.1.1	Bed railings from Patient Room A	bed railings	Patient room	ECU	09 - September
150826658567.1.1	Serum and gas support from Patient Room 1	serum/gas support	Patient room	ECU	09 - September
150826658568.1.1	Bed railings from Patient Room B	bed railings	Patient room	ECU	09 - September
150826658569.1.1	Serum and gas support from Patient Room 2	serum/gas support	Patient room	ECU	09 - September
150826658570.1.1	Bed railings from Patient Room A	bed railings	Patient room	ECU	09 - September
150826658571.1.1	Serum and gas support from Patient Room 3	serum/gas support	Patient room	ECU	09 - September
150826658572.1.1	Dispenser, water tap and flush from Patient Bathroom	dispenser/water tap/flush	Patient bathroom	ECU	09 - September
150826658573.1.1	Countertops, computer and phone	countertops/computer/phone	Nurse station	ECU	09 - September
150826658574.1.1	Dispenser and water tap	dispenser/water tap	Medicament room	ECU	09 - September
150826658575.1.1	Countertops and surfaces	countertops/surfaces	Medicament room	ECU	09 - September
150826658576.1.1	Chairs	chair	Medication room	ECU	09 - September
150826658577.1.1	Serum and gas support	serum/gas support	Medication room	ECU	09 - September
150826658578.1.1	Computers	equipment of common use	Prescription room	ECU	09 - September
150826658579.1.1	Sink	sink	Special procedures room	ECU	09 - September
150826658580.1.1	Bed	bed	Special procedures room	ECU	09 - September
150826658581.1.1	Countertops and surfaces	countertops/surfaces	Bandage room	ECU	09 - Septenber
150826658582.1.1	Bed	bed	Clinic room	ECU	09 - September
150826658583.1.1	Computer table	table	Clinic room	ECU	09 - September
150826658584.1.1	Dispenser and water tap	dispenser/water tap	Clinic room	ECU	09 - September

150826658585.1.1	Chairs	chair	Reception	ECU	09 - September
150826658586.1.1	Table, countertops and domestic appliances	table/countertops/domestic appliances	Lunch room	ECU	09 - September
150826658587.1.1	Bed, table and chair	bed/table/chair	Resting room	ECU	09 - September
150826658588.1.1	Dispenser from the Hall	dispenser	Common place	ECU	09 - September
150826658589.1.1	Medication cart	medical device	Common place	ECU	09 - September

Table S2. Significance of sample grouping on the overall bacterial community structure based on the ANOSIM statistical method. Month, Facility, Room, and Sample-Type represent the four categories tested. Abbreviations: ANOSIM, analysis of similarity. R, correlation coefficient.

	**Bray-Curtis**		**Sorensen-Dice**	
	**R**	**p-value**	**R**	**p-value**
Month	0,08	0,01	0,08	0,01
Facility	0,04	0,01	0,04	0,01
Room	0,1	0,01	0,1	0,01
Sample-Type	0,11	0,01	0,1	0,01

Table S3. Mantel test for the correlation between environmental parameters and samples.

	**Bray-Curtis**		**Sorensen-Dice**	
**Environmental parameters**	**Mantel r**	**p-value**	**Mantel r**	**p-value**
Ambient Temperature	0,01	0,24	0,01	0,38
Surface Temperature	0,01	0,53	0,01	0,55
Relative Humidity	0,01	0,71	0,01	0,95

Table S4. List of the 347 unique OTUs with their respective number of reads.

n. of reads	OTU	Species
50902	541137	*Pseudomonas cremoricolorata*
26321	569900	*Planomicrobium okeanokoites*
17773	590838	*Azotobacter chroococcum*
15505	539446	*Psychrobacter sanguinis*
7748	610016	*Cronobacter turicensis*
5135	592170	*Acinetobacter bereziniae*
4103	560590	*Rhizobium aggregatum*
3159	590735	*Lelliottia amnigena*
3145	545589	*Naxibacter indica*
3104	599439	*Lysinibacillus fusiformis*
3040	577367	*Paenarthrobacter ureafaciens*
2960	615166	*Elizabethkingia meningoseptica*
2729	567508	*Acinetobacter calcoaceticus*
2587	573755	*Pantoea wallisii*
2505	573645	*Paenisporosarcina quisquiliarum*
2442	619733	*Lactobacillus helveticus*
2316	594700	*Massilia varians*
2089	582154	*Brevundimonas terrae*
2056	611186	*Flavobacterium phragmitis*
2039	547383	*Arthrobacter globiformis*
1983	571581	*Acetobacter orientalis*
1943	622445	*Pseudomonas poae*
1806	586536	*Pantoea ananatis*
1777	591385	*Terribacillus saccharophilus*
1743	605087	*Leuconostoc pseudomesenteroides*

1600	595595	*Enterobacter ludwigii*
1549	610754	*Acinetobacter beijerinckii*
1493	622852	*Lactobacillus rhamnosus*
1415	569560	*Bacillus vietnamensis*
1312	574226	*Bacillus aquimaris*
1250	586694	*Asaia bogorensis*
1186	579745	*Psychrobacter celer*
1114	564189	*Pseudomonas jessenii*
1108	593218	*Atlantibacter hermannii*
1084	556296	*Stenotrophomonas maltophilia*
1001	570801	*Erwinia aphidicola*
1001	613007	*Pantoea vagans*
991	619885	*Corynebacterium nuruki*
969	613203	*Pantoea vagans*
909	561855	*Eubacterium limosum*
902	618246	*Corynebacterium simulans*
897	612418	*[Clostridium] innocuum*
813	562635	*Paenibacillus borealis*
796	594654	*Chryseobacterium indoltheticum*
766	593061	*Acinetobacter junii*
750	618890	*Citrobacter freundii*
743	579043	*Acinetobacter marinus*
721	570992	*Erwinia aphidicola*
711	624356	*Achromobacter xylosoxidans*
701	592363	*Bacillus simplex*
694	544670	*Serratia ureilytica*
648	580691	*Bacillus niabensis*
643	555547	*Acinetobacter towneri*
642	624358	*Bacillus simplex*
598	618369	*Serratia proteamaculans*

586	587860	*Bacillus niacini*
577	583646	*Chryseobacterium hispanicum*
571	572207	*Klugiella xanthotipulae*
569	592225	*Sphingobium limneticum*
537	577364	*Pseudarthrobacter equi*
521	571496	*Pluralibacter gergoviae*
521	545503	*Glutamicibacter arilaitensis*
521	617582	*Stenotrophomonas maltophilia*
518	593048	*Stenotrophomonas chelatiphaga*
499	539149	*Pantoea ananatis*
494	552190	*Pseudomonas migulae*
472	618466	*Fusobacterium mortiferum*
455	623021	*Serratia liquefaciens*
428	555209	*Paenibacillus graminis*
426	539750	*Enterobacter hormaechei*
424	614318	*Pseudarthrobacter chlorophenolicus*
424	560085	*Erwinia billingiae*
423	624727	*Campylobacter hominis*
421	554616	*Chryseobacterium pallidum*
418	585797	*Psychrobacter pulmonis*
403	543861	*Elizabethkingia meningoseptica*
394	593491	*Pseudomonas stutzeri*
388	585232	*Stenotrophomonas rhizophila*
385	554463	*Massilia brevitalea*
367	595014	*Psychrobacter meningitidis*
335	609922	*Bacteroides eggerthii*
317	599498	*Paenibacillus lautus*
313	608292	*Aeromonas hydrophila*
300	557030	*Weissella fabaria*
299	618760	*Acinetobacter sp.*

292	592408	*Bacillus flexus*
292	594253	*Ochrobactrum tritici*
275	576428	*Pseudomonas composti*
273	600621	*Fusobacterium mortiferum*
272	614559	*Klebsiella oxytoca*
270	587762	*Deinococcus grandis*
249	598146	*Rahnella aquatilis*
247	551122	*Aeromonas caviae*
243	615022	*Capnocytophaga sputigena*
243	582462	*Bacillus massiliosenegalensis*
229	618394	*Actinomyces sp.*
211	550840	*Acinetobacter towneri*
207	624463	*Sphingobium yanoikuyae*
185	543013	*Acinetobacter johnsonii*
184	624263	*Tetragenococcus halophilus*
183	622649	*Raoultella ornithinolytica*
182	582714	*Gluconobacter frateurii*
179	591785	*Glutamicibacter arilaitensis*
176	613062	*Weissella fabalis*
165	622269	*Streptococcus agalactiae*
161	572002	*Pseudomonas oryzihabitans*
157	569657	*Olivibacter jilunii*
144	618621	*Corynebacterium coyleae*
136	569925	*Lactobacillus casei*
135	616434	*Pseudarthrobacter scleromae*
135	618630	*Pseudomonas veronii*
135	544532	*Pseudomonas migulae*
127	618036	*Capnocytophaga gingivalis*
125	595270	*Klebsiella oxytoca*
124	571177	*Massilia aerilata*

111	583551	*Corynebacterium mastitidis*
111	598035	*Aeromonas caviae*
109	596164	*Bacillus benzoevorans*
106	619691	*Raoultella ornithinolytica*
105	615184	*Citrobacter rodentium*
105	552144	*Brevibacterium oceani*
105	622848	*Salmonella enterica*
102	607471	*Scardovia wiggsiae*
101	624241	*Pseudomonas putida*
101	586375	*Alistipes onderdonkii*
98	621566	*Rahnella aquatilis*
88	622294	*Bacillus cereus sp. group*
85	594146	*Arthrobacter pascens*
85	624019	*Pseudomonas putida*
79	617897	*Roseburia inulinivorans*
79	573545	*Veillonella rogosae*
79	586010	*Prevotella nanceiensis*
77	600070	*Advenella kashmirensis*
75	619563	*Oceanobacillus picturae*
69	585625	*Acinetobacter johnsonii*
67	595723	*Facklamia languida*
66	588889	*Morococcus cerebrosus*
66	558387	*Hyalangium minutum*
62	576022	*Sphingomonas dokdonensis*
59	613341	*Pseudomonas marginalis*
58	546076	*Lysinibacillus sphaericus*
57	591996	*Pseudomonas oleovorans*
55	571707	*Auritidibacter ignavus*
55	544133	*Stenotrophomonas maltophilia*
54	611697	*Actinomyces oris*

52	548650	*Sphingobacterium cladoniae*
51	552521	*Methylobacterium jeotgali*
50	580960	*Bacillus megaterium*
49	555159	*Empedobacter falsenii*
49	613570	*Acinetobacter junii*
47	594781	*Ralstonia mannitolilytica*
47	592569	*Pantoea agglomerans*
46	562552	*Pseudomonas rhodesiae*
44	600850	*Burkholderia ambifaria*
44	618392	*Actinomyces odontolyticus*
43	624543	*Pseudomonas nitroreducens*
41	587949	*Acinetobacter guillouiae*
40	596212	*Bacteroides massiliensis*
40	551860	*Prevotella oulorum*
40	616381	*Moraxella lincolnii*
39	566275	*Staphylococcus saccharolyticus*
39	611270	*Fusicatenibacter saccharivorans*
39	599232	*Dyadobacter soli*
38	611388	*Acinetobacter junii*
37	572770	*Rhizobium daejeonense*
36	588048	*Lactobacillus casei*
35	607852	*Brochothrix thermosphacta*
35	552585	*Cobetia crustatorum*
35	538465	*Aeromonas caviae*
34	619224	*Lactobacillus delbrueckii*
32	580143	*Pseudomonas panipatensis*
31	608486	*Lachnoanaerobaculum saburreum*
30	590871	*Lactococcus piscium*
30	623044	*Arcobacter butzleri*
30	562346	*Corynebacterium minutissimum*

30	600999	*Leptotrichia wadei*
30	614399	*Citrobacter werkmanii*
30	574263	*Solibacillus isronensis*
29	576273	*Luteibacter rhizovicinus*
29	602482	*Acinetobacter parvus*
29	548596	*Corynebacterium coyleae*
29	578939	*Raoultella ornithinolytica*
29	561660	*Sphingobium estrogenivorans*
28	608836	*Lactobacillus iners*
27	586962	*Anoxybacillus flavithermus*
27	600958	*Prevotella disiens*
27	614912	*Raoultella ornithinolytica*
27	558128	*Kaistia geumhonensis*
26	624244	*Bifidobacterium longum*
26	594207	*Paenibacillus amylolyticus*
26	559815	*Stenotrophomonas maltophilia*
25	603885	*Burkholderia multivorans*
25	562191	*Corynebacterium segmentosum*
24	609905	*[Eubacterium] siraeum*
23	624460	*Mesorhizobium huakuii*
23	562556	*Pseudomonas rhodesiae*
23	565437	*Facklamia ignava*
23	581613	*Sphingomonas dokdonensis*
23	592259	*Kurthia zopfii*
22	574496	*Rhodococcus artemisiae*
22	557235	*Staphylococcus carnosus*
22	616774	*Brevibacterium epidermidis*
21	571700	*Micrococcus terreus*
21	591451	*Paeniclostridium ghonii*
21	579661	*Sediminibacterium salmoneum*

21	583573	*Sphingomonas yunnanensis*
20	605579	*[Eubacterium] eligens*
20	578689	*Comamonas aquatica*
20	589558	*Acinetobacter haemolyticus*
20	552602	*Skermanella aerolata*
20	607799	*Brachybacterium faecium*
20	544919	*Prevotella melaninogenica*
20	587975	*Tatumella punctata*
20	598566	*Bacteroides vulgatus*
20	611300	*Methylobacterium komagatae*
19	622892	*Lactobacillus acidophilus*
19	611232	*Methylobacterium aquaticum*
19	615892	*Leuconostoc lactis*
19	564973	*Corynebacterium riegelii*
18	616880	*Dermacoccus profundi*
18	617396	*Shewanella putrefaciens*
18	617681	*Melittangium boletus*
18	619346	*Pantoea agglomerans*
18	599810	*Neisseria sicca*
18	623513	*Paracoccus kocurii*
17	584581	*Selenomonas noxia*
17	602393	*Citrobacter freundii*
17	612823	*Peptoniphilus coxii*
17	618874	*Rhodococcus fascians*
17	615628	*Neisseria perflava*
17	615748	*Bifidobacterium adolescentis*
17	599714	*Neisseria cinerea*
16	556253	*Rhizobium gallicum*
16	561365	*Rahnella aquatilis*
16	572041	*Parasegetibacter luojiensis*

16	577010	*Veillonella denticariosi*
16	581684	*Rhizobium sp.*
16	616914	*Streptococcus sinensis*
15	617730	*Campylobacter concisus*
15	622191	*Streptococcus intermedius*
15	617098	*Megasphaera micronuciformis*
15	567953	*Pseudoglutamicibacter cumminsii*
15	539822	*Bacteroides uniformis*
15	572437	*Stenotrophomonas maltophilia*
15	587173	*Rothia aeria*
14	569538	*Weissella hellenica*
14	619012	*Phenylobacterium haematophilum*
14	622476	*Cronobacter sakazakii*
14	574833	*Paenibacillus hunanensis*
14	617118	*Streptococcus equinus*
13	567613	*Leclercia adecarboxylata*
13	585487	*Sporosarcina thermotolerans*
13	624357	*Bosea thiooxidans*
13	608881	*Veillonella parvula*
13	541118	*Sphingomonas yabuuchiae*
13	572147	*Sphingomonas hunanensis*
13	551585	*Kingella denitrificans*
13	591711	*Bacteroides fragilis*
13	603663	*Neisseria bacilliformis*
12	550955	*Brevundimonas aurantiaca*
12	559028	*Cupriavidus pauculus*
12	576137	*Mycoplasma amphoriforme*
12	586262	*Chryseobacterium solincola*
12	622157	*Leuconostoc gelidum*
12	622198	*Streptococcus anginosus*

12	624978	*uncultured Clostridium sp.*
11	538401	*Pseudoclavibacter bifida*
11	589760	*Rickettsia endosymbiont of Bemisia tabaci*
11	597034	*Pedobacter antarcticus*
11	598118	*Parabacteroides goldsteinii*
11	568757	*Alloiococcus otitis*
11	623182	*Enterococcus faecium*
11	541958	*Sphingomonas oryziterrae*
11	620978	*Bacillus coagulans*
11	563179	*Fusobacterium simiae*
11	577941	*Haemophilus parahaemolyticus*
10	571036	*Cobetia marina*
10	591462	*Helcococcus seattlensis*
10	596253	*Faecalibacterium prausnitzii*
10	578222	*Kocuria rhizophila*
10	586187	*Brevundimonas kwangchunensis*
10	617277	*Cellulosimicrobium cellulans*
10	598433	*Blastococcus aggregatus*
10	537940	*Haemophilus parahaemolyticus*
10	568380	*Corynebacterium durum*
10	577023	*Sediminibacterium salmoneum*
10	583027	*Streptomyces radiopugnans*
9	552654	*Geodermatophilus obscurus*
9	571333	*Staphylococcus saprophyticus*
9	580777	*Actinomyces naeslundii*
9	618864	*Caulobacter sp.*
9	599805	*Selenomonas noxia*
9	620919	*Streptococcus lutetiensis*
9	575747	*Rhizorhapis suberifaciens*
9	604819	*Rheinheimera perlucida*

9	615366	*Bosea minatitlanensis*
9	550894	*Achromobacter xylosoxidans*
9	566808	*Acinetobacter johnsonii*
9	604189	*Parvimonas micra*
9	608511	*Eremococcus coleocola*
9	618875	*Bacillus licheniformis*
8	608416	*Streptococcus anginosus*
8	617624	*Caulobacter mirabilis*
8	559422	*Prevotella timonensis*
8	616544	*Lactobacillus jensenii*
8	539049	*Moraxella atlantae*
8	582745	*Macrococcus brunensis*
8	593664	*Xanthobacter flavus*
8	576222	*Corynebacterium aurimucosum*
8	600865	*Acinetobacter ursingii*
7	565772	*Desemzia incerta*
7	573780	*Cellulomonas marina*
7	579592	*Sphingopyxis ummariensis*
7	600174	*Oribacterium sinus*
7	615023	*Oligella urethralis*
7	618486	*Ralstonia pickettii*
7	623755	*Campylobacter gracilis*
7	559005	*Afipia genosp. 13*
7	554268	*Enterobacter cloacae*
7	595571	*Enterobacter cloacae*
7	540944	*Zoogloea resiniphila*
7	549389	*Tatumella punctata*
7	618758	*Filifactor alocis*
6	548444	*Sphingomonas rosea*
6	555709	*Enterococcus avium*

6	558269	*Streptococcus sobrinus*
6	615731	*Sphingomonas canadensis*
6	621950	*Fusobacterium nucleatum*
6	539305	*[Clostridium] hiranonis*
6	561203	*Sphingopyxis macrogoltabida*
6	569972	*Novosphingobium subterraneum*
6	571079	*Pelomonas saccharophila*
6	594851	*Pseudomonas putida*
6	600210	*Lactobacillus jensenii*
6	609392	*Staphylococcus lugdunensis*
6	615333	*Enterococcus malodoratus*
6	617762	*Streptococcus sobrinus*
6	580579	*Bacillus firmus*
6	582871	*Brevundimonas kwangchunensis*
6	614295	*Moraxella atlantae*
6	617611	*Kocuria rhizophila*
6	583487	*Achromobacter xylosoxidans*
6	601676	*Prevotella salivae*
5	554704	*Staphylococcus epidermidis*
5	590164	*Bacillus cibi*
5	561895	*Corynebacterium afermentans*
5	570018	*Sphingomonas jaspsi*

Table S5. Pathogen status of the 70 taxa present in more than 5% of the samples.

Species	OTU	Pathogen Status	Prevalence (%)	Degree	Closeness Centrality	Betweeness Centrality	Clustering Coefficient	Eigen Centrality
Acinetobacter baumannii	610300	nosocomial pathogen	15	9	0,46	52,18	0,01	0,07
Acinetobacter baumannii	624096	nosocomial pathogen	30	5	0	0	0,15	0,28
Acinetobacter nosocomialis	606882	nosocomial pathogen	5	21	0,56	148,87	0,04	0,09
Acinetobacter nosocomialis	607150	nosocomial pathogen	5	4	0,58	1	0,08	0
Bacillus cereus sp. group	624484	nosocomial pathogen	5	8	0	0	0,04	0,37
Escherichia coli	624449	nosocomial pathogen	38	7	0	0	0,02	0,26
Klebsiella oxytoca	621287	nosocomial pathogen	15	4	1	3,45	0,08	0,08
Klebsiella oxytoca	621896	nosocomial pathogen	5	6	0,5	7,42	0,2	0,13
Klebsiella pneumoniae	607520	nosocomial pathogen	10	9	0,42	22,9	0,06	0,02
Klebsiella pneumoniae	620558	nosocomial pathogen	15	12	0,54	54,86	0,11	0,1
Klebsiella pneumoniae	620675	nosocomial pathogen	5	4	1	3,83	0	0,03
Klebsiella pneumoniae	623195	nosocomial pathogen	20	9	0,8	14,6	0,07	0,14
Klebsiella pneumoniae	623210	nosocomial pathogen	15	8	0,75	21,07	0,11	0,35
Pseudomonas aeruginosa	624114	nosocomial pathogen	10	14	0	0	0,05	0,56
Pseudomonas putida	623836	nosocomial pathogen	20	5	1	2,98	0,15	0,07
Pseudomonas putida	624672	nosocomial pathogen	25	7	0	0	0,1	0,61
Staphylococcus capitis	605239	nosocomial pathogen	10	0	0	0	0	0
Staphylococcus epidermidis	623550	nosocomial pathogen	35	1	0	0	0	0,09
Staphylococcus haemolyticus	591357	nosocomial pathogen	5	16	0,56	49,1	0,08	0,02
Staphylococcus haemolyticus	624535	nosocomial pathogen	10	4	0	0	0	0,15
Stenotrophomonas maltophilia	618830	nosocomial pathogen	5	6	0,46	18,23	0,03	0,09
Acinetobacter johnsonii	561250	rare nosocomial opportunistic pathogen	5	4	0,39	0	0	0
Acinetobacter johnsonii	570673	rare nosocomial opportunistic pathogen	5	5	0,39	3,23	0,05	0
Acinetobacter johnsonii	583122	rare nosocomial opportunistic pathogen	5	9	0,48	0	0,06	0
Acinetobacter johnsonii	592001	rare nosocomial opportunistic pathogen	15	2	0,36	0	0	0
Acinetobacter johnsonii	602399	rare nosocomial opportunistic pathogen	10	9	0,4	45,49	0,03	0,06
Acinetobacter johnsonii	613197	rare nosocomial opportunistic pathogen	10	8	0,49	11,83	0,05	0,03

Acinetobacter lwoffii	614684	rare nosocomial opportunistic pathogen	15	4	0,7	0	0,08	0
Acinetobacter ursingii	602718	rare nosocomial opportunistic pathogen	15	9	0,47	32,99	0,03	0,02
Acinetobacter ursingii	619120	rare nosocomial opportunistic pathogen	15	3	1	2	0,17	0
Pseudomonas oryzihabitans	611399	rare nosocomial opportunistic pathogen	15	8	0,49	11,7	0,13	0,02
Serratia marcescens	622311	rare nosocomial opportunistic pathogen	5	10	0,58	21,71	0,07	0,27
Serratia marcescens	623929	rare nosocomial opportunistic pathogen	5	14	0,75	35,52	0,09	0,4
Staphylococcus hominis	598572	rare nosocomial opportunistic pathogen	15	3	0,42	0,91	0	0,02
Streptococcus mitis	624183	rare nosocomial opportunistic pathogen	20	4	1	16,95	0	0,37
Acinetobacter radioresistens	613572	opportunistic pathogen	5	7	0,45	15,35	0,1	0,12
Finegoldia magna	618642	opportunistic pathogen	5	7	0,53	20,45	0,05	0,1
Fusobacterium nucleatum	611105	opportunistic pathogen	5	9	0,48	43,03	0,07	0,11
Haemophilus parainfluenzae	608415	opportunistic pathogen	5	6	1	4,48	0,13	0,04
Staphylococcus warneri	621868	opportunistic pathogen	5	11	0,56	66,5	0,05	0,21
Anaerococcus vaginalis	617614	rare opportunistic pathogen	5	4	0,45	15,82	0	0,06
Corynebacterium tuberculostearicum	562774	rare opportunistic pathogen	10	12	0,48	0	0,11	0
Fusobacterium periodonticum	600153	rare opportunistic pathogen	5	7	0,49	30,71	0,1	0,02
Granulicatella adiacens	600022	rare opportunistic pathogen	5	7	0,35	26	0,05	0,03
Massilia timonae	595823	rare opportunistic pathogen	5	7	0,41	6,37	0,07	0
Methylobacterium radiotolerans	623831	rare opportunistic pathogen	10	4	0	0	0,08	0,2
Moraxella osloensis	592450	rare opportunistic pathogen	20	12	0,47	24,73	0,07	0,01
Murdochiella asaccharolytica	587128	rare opportunistic pathogen	5	3	0	0	0,17	0,14
Pantoea dispersa	599261	rare opportunistic pathogen	5	10	0,51	20,14	0,13	0,02
Pantoea dispersa	619869	rare opportunistic pathogen	10	8	0,5	17,87	0,13	0,07
Peptoniphilus asaccharolyticus	565732	rare opportunistic pathogen	5	7	0,48	0	0,07	0
Pseudomonas fluorescens	624028	rare opportunistic pathogen	5	8	1	35,2	0,05	0,45
Pseudomonas stutzeri	596827	rare opportunistic pathogen	20	5	0,4	0	0,1	0
Pseudomonas stutzeri	620673	rare opportunistic pathogen	20	4	0,67	3,83	0,08	0,02
Pseudomonas stutzeri	621004	rare opportunistic pathogen	5	9	0,63	70,62	0,03	0,2
Pseudomonas stutzeri	621954	rare opportunistic pathogen	5	16	0,73	90,68	0,1	0,36
Staphylococcus cohnii	598217	rare opportunistic pathogen	5	13	0,53	16,3	0,06	0,01
Staphylococcus cohnii	623865	rare opportunistic pathogen	5	12	1	17,2	0,1	0,8
Streptococcus parasanguinis	608270	rare opportunistic pathogen	5	6	0,46	23,72	0,07	0,04

Streptococcus salivarius	620690	rare opportunistic pathogen	5	11	0,45	45,74	0,08	0,17
Agrobacterium tumefaciens	617866	non pathogen	5	9	0,63	19,36	0,06	0,07
Agrobacterium tumefaciens	624660	non pathogen	5	4	0	0	0,08	0,1
Bacillus megaterium	624482	non pathogen	5	7	1	1,75	0,1	0,17
Cloacibacterium normanense	542269	non pathogen	5	11	0,48	0	0,08	0
Paracoccus carotinifaciens	589152	non pathogen	10	11	0,41	23,19	0,04	0,01
Porphyromonas bennonis	584857	non pathogen	5	3	0	0	0,33	0,13
Pseudomonas monteilii	624642	non pathogen	10	15	0	0	0,08	1
Pseudomonas plecoglossicida	623690	non pathogen	5	10	0,55	20,76	0,08	0,29
Rubrobacter xylanophilus	600454	non pathogen	10	11	0,49	59,53	0,04	0,03
Sphingobium yanoikuyae	623802	non pathogen	5	11	0,67	6,87	0,12	0,56

Table S6. Interaction patterns of the 70 taxa present in more than 5% of the samples. n=negative correlation; p= positive correlation

Source		Target		Corr
**Species**	**OTU**	**Species**	**OTU**	
Acinetobacter baumannii	610300	*Fusobacterium nucleatum*	611105	n
Acinetobacter baumannii	610300	*Serratia marcescens*	622311	n
Acinetobacter baumannii	610300	*Stenotrophomonas maltophilia*	618830	n
Acinetobacter baumannii	610300	*Streptococcus salivarius*	620690	n
Acinetobacter baumannii	610300	*Staphylococcus cohnii*	623865	p
Acinetobacter johnsonii	592001	*Acinetobacter baumannii*	610300	n
Acinetobacter johnsonii	613197	*Agrobacterium tumefaciens*	624660	n
Acinetobacter johnsonii	583122	*Bacillus megaterium*	624482	n
Acinetobacter johnsonii	583122	*Finegoldia magna*	618642	n
Acinetobacter johnsonii	583122	*Fusobacterium nucleatum*	611105	n
Acinetobacter johnsonii	561250	*Fusobacterium periodonticum*	600153	n
Acinetobacter johnsonii	570673	*Granulicatella adiacens*	600022	n
Acinetobacter johnsonii	602399	*Klebsiella oxytoca*	621287	n
Acinetobacter johnsonii	592001	*Klebsiella pneumoniae*	607520	n
Acinetobacter johnsonii	583122	*Pantoea dispersa*	619869	n
Acinetobacter johnsonii	561250	*Pseudomonas aeruginosa*	624114	n
Acinetobacter johnsonii	613197	*Serratia marcescens*	623929	n
Acinetobacter johnsonii	613197	*Staphylococcus cohnii*	623865	n
Acinetobacter johnsonii	570673	*Staphylococcus haemolyticus*	591357	n
Acinetobacter johnsonii	613197	*Staphylococcus warneri*	621868	n
Acinetobacter johnsonii	583122	*Stenotrophomonas maltophilia*	618830	n
Acinetobacter johnsonii	613197	*Stenotrophomonas maltophilia*	618830	n
Acinetobacter johnsonii	602399	*Streptococcus parasanguinis*	608270	n
Acinetobacter johnsonii	583122	*Streptococcus salivarius*	620690	n
Acinetobacter johnsonii	602399	*Acinetobacter radioresistens*	613572	p
Acinetobacter johnsonii	561250	*Agrobacterium tumefaciens*	624660	p
Acinetobacter johnsonii	583122	*Bacillus cereus sp. group*	624484	p

Acinetobacter johnsonii	570673	*Bacillus megaterium*	624482	p
Acinetobacter johnsonii	613197	*Finegoldia magna*	618642	p
Acinetobacter johnsonii	583122	*Haemophilus parainfluenzae*	608415	p
Acinetobacter johnsonii	602399	*Pantoea dispersa*	619869	p
Acinetobacter johnsonii	602399	*Pseudomonas fluorescens*	624028	p
Acinetobacter johnsonii	561250	*Serratia marcescens*	622311	p
Acinetobacter johnsonii	583122	*Sphingobium yanoikuyae*	623802	p
Acinetobacter johnsonii	570673	*Staphylococcus warneri*	621868	p
Acinetobacter lwoffii	614684	*Acinetobacter ursingii*	619120	n
Acinetobacter lwoffii	614684	*Pseudomonas putida*	623836	n
Acinetobacter lwoffii	614684	*Acinetobacter baumannii*	624096	p
Acinetobacter lwoffii	614684	*Pseudomonas putida*	624672	p
Acinetobacter nosocomialis	606882	*Acinetobacter baumannii*	624096	n
Acinetobacter nosocomialis	606882	*Acinetobacter radioresistens*	613572	n
Acinetobacter nosocomialis	606882	*Agrobacterium tumefaciens*	624660	n
Acinetobacter nosocomialis	607150	*Klebsiella pneumoniae*	623195	n
Acinetobacter nosocomialis	606882	*Methylobacterium radiotolerans*	623831	n
Acinetobacter nosocomialis	607150	*Pseudomonas aeruginosa*	624114	n
Acinetobacter nosocomialis	606882	*Pseudomonas putida*	623836	n
Acinetobacter nosocomialis	606882	*Pseudomonas putida*	624672	n
Acinetobacter nosocomialis	607150	*Staphylococcus haemolyticus*	624535	n
Acinetobacter nosocomialis	606882	*Acinetobacter baumannii*	610300	p
Acinetobacter nosocomialis	606882	*Finegoldia magna*	618642	p
Acinetobacter nosocomialis	606882	*Murdochiella asaccharolytica*	587128	p
Acinetobacter nosocomialis	606882	*Porphyromonas bennonis*	584857	p
Acinetobacter nosocomialis	606882	*Pseudomonas monteilii*	624642	p
Acinetobacter nosocomialis	606882	*Pseudomonas plecoglossicida*	623690	p
Acinetobacter nosocomialis	606882	*Pseudomonas stutzeri*	621004	p
Acinetobacter nosocomialis	606882	*Serratia marcescens*	617614	p
Acinetobacter nosocomialis	606882	*Streptococcus mitis*	624183	p
Acinetobacter radioresistens	613572	*Murdochiella asaccharolytica*	587128	n
Acinetobacter radioresistens	613572	*Pseudomonas stutzeri*	621004	p

Acinetobacter radioresistens	613572	*Staphylococcus cohnii*	623865	p
Acinetobacter ursingii	602718	*Acinetobacter baumannii*	624096	n
Acinetobacter ursingii	619120	*Bacillus megaterium*	624482	n
Acinetobacter ursingii	602718	*Pseudomonas stutzeri*	620673	n
Acinetobacter ursingii	602718	*Serratia marcescens*	622311	n
Acinetobacter ursingii	602718	*Staphylococcus warneri*	621868	n
Acinetobacter ursingii	602718	*Acinetobacter nosocomialis*	606882	p
Acinetobacter ursingii	602718	*Escherichia coli*	624449	p
Acinetobacter ursingii	602718	*Pseudomonas aeruginosa*	624114	p
Acinetobacter ursingii	619120	*Pseudomonas monteilii*	624642	p
Agrobacterium tumefaciens	617866	*Pseudomonas plecoglossicida*	623690	n
Agrobacterium tumefaciens	617866	*Sphingobium yanoikuyae*	623802	n
Agrobacterium tumefaciens	617866	*Staphylococcus haemolyticus*	624535	n
Agrobacterium tumefaciens	617866	*Pseudomonas fluorescens*	624028	p
Agrobacterium tumefaciens	617866	*Pseudomonas putida*	624672	p
Agrobacterium tumefaciens	617866	*Serratia marcescens*	623929	p
Bacillus megaterium	624482	*Pseudomonas monteilii*	624642	p
Cloacibacterium normanense	542269	*Acinetobacter nosocomialis*	607150	n
Cloacibacterium normanense	542269	*Klebsiella pneumoniae*	607520	n
Cloacibacterium normanense	542269	*Serratia marcescens*	623929	n
Cloacibacterium normanense	542269	*Staphylococcus haemolyticus*	591357	n
Cloacibacterium normanense	542269	*Acinetobacter baumannii*	610300	p
Cloacibacterium normanense	542269	*Acinetobacter johnsonii*	570673	p
Cloacibacterium normanense	542269	*Moraxella osloensis*	592450	p
Cloacibacterium normanense	542269	*Paracoccus carotinifaciens*	589152	p
Cloacibacterium normanense	542269	*Pseudomonas aeruginosa*	624114	p
Cloacibacterium normanense	542269	*Pseudomonas monteilii*	624642	p
Cloacibacterium normanense	542269	*Rubrobacter xylanophilus*	600454	p
Corynebacterium tuberculostearicum	562774	*Bacillus megaterium*	624482	n
Corynebacterium tuberculostearicum	562774	*Granulicatella adiacens*	600022	n

Corynebacterium tuberculostearicum	562774	*Haemophilus parainfluenzae*	608415	n
Corynebacterium tuberculostearicum	562774	*Klebsiella oxytoca*	621896	n
Corynebacterium tuberculostearicum	562774	*Pantoea dispersa*	599261	n
Corynebacterium tuberculostearicum	562774	*Pseudomonas monteilii*	624642	n
Corynebacterium tuberculostearicum	562774	*Fusobacterium nucleatum*	611105	p
Corynebacterium tuberculostearicum	562774	*Klebsiella pneumoniae*	623210	p
Corynebacterium tuberculostearicum	562774	*Moraxella osloensis*	592450	p
Corynebacterium tuberculostearicum	562774	*Paracoccus carotinifaciens*	589152	p
Corynebacterium tuberculostearicum	562774	*Pseudomonas stutzeri*	621954	p
Corynebacterium tuberculostearicum	562774	*Serratia marcescens*	623929	p
Finegoldia magna	618642	*Klebsiella pneumoniae*	623195	n
Finegoldia magna	618642	*Porphyromonas bennonis*	584857	p
Finegoldia magna	618642	*Serratia marcescens*	623929	p
Fusobacterium nucleatum	611105	*Serratia marcescens*	623929	n
Fusobacterium nucleatum	611105	*Pseudomonas plecoglossicida*	623690	p
Fusobacterium nucleatum	611105	*Pseudomonas stutzeri*	621004	p
Fusobacterium periodonticum	600153	*Klebsiella pneumoniae*	620558	n
Fusobacterium periodonticum	600153	*Pseudomonas stutzeri*	621954	n
Fusobacterium periodonticum	600153	*Serratia marcescens*	623929	n
Fusobacterium periodonticum	600153	*Stenotrophomonas maltophilia*	618830	n
Granulicatella adiacens	600022	*Escherichia coli*	624449	n
Granulicatella adiacens	600022	*Methylobacterium radiotolerans*	623831	n
Granulicatella adiacens	600022	*Rubrobacter xylanophilus*	600454	n
Granulicatella adiacens	600022	*Pseudomonas monteilii*	624642	p
Haemophilus parainfluenzae	608415	*Bacillus cereus sp. group*	624484	n

Haemophilus parainfluenzae	608415	*Pseudomonas monteilii*	624642	n
Klebsiella oxytoca	621287	*Escherichia coli*	624449	n
Klebsiella oxytoca	621896	*Klebsiella pneumoniae*	623210	p
Klebsiella oxytoca	621287	*Pseudomonas aeruginosa*	624114	p
Klebsiella oxytoca	621896	*Pseudomonas stutzeri*	621954	p
Klebsiella pneumoniae	607520	*Bacillus cereus sp. group*	624484	n
Klebsiella pneumoniae	620675	*Escherichia coli*	624449	n
Klebsiella pneumoniae	607520	*Murdochiella asaccharolytica*	587128	n
Klebsiella pneumoniae	620558	*Pseudomonas aeruginosa*	624114	n
Klebsiella pneumoniae	623195	*Staphylococcus cohnii*	623865	n
Klebsiella pneumoniae	623210	*Streptococcus mitis*	624183	n
Klebsiella pneumoniae	620675	*Bacillus cereus sp. group*	624484	p
Klebsiella pneumoniae	620558	*Bacillus megaterium*	624482	p
Klebsiella pneumoniae	607520	*Fusobacterium nucleatum*	611105	p
Klebsiella pneumoniae	607520	*Haemophilus parainfluenzae*	608415	p
Klebsiella pneumoniae	623195	*Pseudomonas monteilii*	624642	p
Klebsiella pneumoniae	623210	*Pseudomonas monteilii*	624642	p
Klebsiella pneumoniae	620558	*Pseudomonas stutzeri*	621954	p
Klebsiella pneumoniae	607520	*Serratia marcescens*	623929	p
Klebsiella pneumoniae	623195	*Sphingobium yanoikuyae*	623802	p
Klebsiella pneumoniae	620558	*Staphylococcus cohnii*	623865	p
Klebsiella pneumoniae	620558	*Staphylococcus warneri*	621868	p
Klebsiella pneumoniae	620558	*Streptococcus salivarius*	620690	p
Massilia timonae	595823	*Fusobacterium periodonticum*	600153	n
Massilia timonae	595823	*Klebsiella pneumoniae*	607520	n
Massilia timonae	595823	*Pseudomonas monteilii*	624642	n
Massilia timonae	595823	*Serratia marcescens*	617614	n
Massilia timonae	595823	*Acinetobacter johnsonii*	602399	p
Massilia timonae	595823	*Serratia marcescens*	623929	p
Moraxella osloensis	592450	*Acinetobacter johnsonii*	602399	n
Moraxella osloensis	592450	*Haemophilus parainfluenzae*	608415	n
Moraxella osloensis	592450	*Klebsiella pneumoniae*	620558	n

Moraxella osloensis	592450	*Staphylococcus cohnii*	598217	n
Moraxella osloensis	592450	*Streptococcus salivarius*	620690	n
Moraxella osloensis	592450	*Acinetobacter johnsonii*	613197	p
Moraxella osloensis	592450	*Acinetobacter ursingii*	602718	p
Moraxella osloensis	592450	*Fusobacterium periodonticum*	600153	p
Moraxella osloensis	592450	*Sphingobium yanoikuyae*	623802	p
Moraxella osloensis	592450	*Staphylococcus cohnii*	623865	p
Pantoea dispersa	599261	*Agrobacterium tumefaciens*	617866	n
Pantoea dispersa	619869	*Klebsiella oxytoca*	621287	n
Pantoea dispersa	599261	*Klebsiella pneumoniae*	620558	n
Pantoea dispersa	599261	*Pseudomonas plecoglossicida*	623690	n
Pantoea dispersa	619869	*Pseudomonas putida*	624672	n
Pantoea dispersa	619869	*Pseudomonas stutzeri*	621954	n
Pantoea dispersa	619869	*Sphingobium yanoikuyae*	623802	n
Pantoea dispersa	599261	*Acinetobacter johnsonii*	613197	p
Pantoea dispersa	599261	*Acinetobacter nosocomialis*	606882	p
Pantoea dispersa	599261	*Pseudomonas stutzeri*	621954	p
Pantoea dispersa	599261	*Serratia marcescens*	622311	p
Pantoea dispersa	599261	*Staphylococcus cohnii*	623865	p
Paracoccus carotinifaciens	589152	*Fusobacterium nucleatum*	611105	n
Paracoccus carotinifaciens	589152	*Granulicatella adiacens*	600022	n
Paracoccus carotinifaciens	589152	*Pantoea dispersa*	619869	n
Paracoccus carotinifaciens	589152	*Staphylococcus haemolyticus*	624535	n
Paracoccus carotinifaciens	589152	*Staphylococcus hominis*	598572	n
Paracoccus carotinifaciens	589152	*Acinetobacter johnsonii*	602399	p
Paracoccus carotinifaciens	589152	*Klebsiella pneumoniae*	620558	p
Paracoccus carotinifaciens	589152	*Streptococcus salivarius*	620690	p
Peptoniphilus asaccharolyticus	565732	*Acinetobacter nosocomialis*	606882	n
Peptoniphilus asaccharolyticus	565732	*Massilia timonae*	595823	p
Peptoniphilus asaccharolyticus	565732	*Paracoccus carotinifaciens*	589152	p
Peptoniphilus asaccharolyticus	565732	*Pseudomonas oryzihabitans*	611399	p
Peptoniphilus asaccharolyticus	565732	*Pseudomonas plecoglossicida*	623690	p

Peptoniphilus asaccharolyticus	565732	*Pseudomonas stutzeri*	620673	p
Peptoniphilus asaccharolyticus	565732	*Staphylococcus haemolyticus*	591357	p
Pseudomonas fluorescens	624028	*Pseudomonas aeruginosa*	624114	n
Pseudomonas fluorescens	624028	*Acinetobacter baumannii*	624096	p
Pseudomonas oryzihabitans	611399	*Sphingobium yanoikuyae*	623802	n
Pseudomonas oryzihabitans	611399	*Staphylococcus warneri*	621868	n
Pseudomonas oryzihabitans	611399	*Klebsiella oxytoca*	621896	p
Pseudomonas oryzihabitans	611399	*Klebsiella pneumoniae*	623195	p
Pseudomonas oryzihabitans	611399	*Klebsiella pneumoniae*	623210	p
Pseudomonas oryzihabitans	611399	*Streptococcus salivarius*	620690	p
Pseudomonas plecoglossicida	623690	*Staphylococcus cohnii*	623865	n
Pseudomonas plecoglossicida	623690	*Serratia marcescens*	623929	p
Pseudomonas putida	623836	*Acinetobacter baumannii*	624096	n
Pseudomonas putida	623836	*Bacillus cereus sp. group*	624484	p
Pseudomonas stutzeri	596827	*Klebsiella pneumoniae*	607520	n
Pseudomonas stutzeri	621954	*Klebsiella pneumoniae*	623210	n
Pseudomonas stutzeri	621954	*Pseudomonas fluorescens*	624028	n
Pseudomonas stutzeri	596827	*Staphylococcus cohnii*	623865	n
Pseudomonas stutzeri	621004	*Staphylococcus haemolyticus*	624535	n
Pseudomonas stutzeri	596827	*Acinetobacter baumannii*	610300	p
Pseudomonas stutzeri	596827	*Acinetobacter ursingii*	602718	p
Pseudomonas stutzeri	621004	*Escherichia coli*	624449	p
Pseudomonas stutzeri	620673	*Klebsiella pneumoniae*	620675	p
Pseudomonas stutzeri	621004	*Klebsiella pneumoniae*	623210	p
Pseudomonas stutzeri	620673	*Pseudomonas aeruginosa*	624114	p
Pseudomonas stutzeri	621954	*Pseudomonas aeruginosa*	624114	p
Pseudomonas stutzeri	621004	*Pseudomonas fluorescens*	624028	p
Pseudomonas stutzeri	621954	*Pseudomonas monteilii*	624642	p
Pseudomonas stutzeri	596827	*Pseudomonas plecoglossicida*	623690	p
Pseudomonas stutzeri	621954	*Pseudomonas putida*	624672	p
Pseudomonas stutzeri	621954	*Serratia marcescens*	622311	p
Pseudomonas stutzeri	621954	*Sphingobium yanoikuyae*	623802	p

Pseudomonas stutzeri	621954	*Staphylococcus cohnii*	623865	p
Pseudomonas stutzeri	621004	*Staphylococcus epidermidis*	623550	p
Rubrobacter xylanophilus	600454	*Acinetobacter johnsonii*	602399	n
Rubrobacter xylanophilus	600454	*Acinetobacter nosocomialis*	606882	n
Rubrobacter xylanophilus	600454	*Serratia marcescens*	623929	n
Rubrobacter xylanophilus	600454	*Agrobacterium tumefaciens*	624660	p
Rubrobacter xylanophilus	600454	*Fusobacterium nucleatum*	611105	p
Rubrobacter xylanophilus	600454	*Klebsiella pneumoniae*	623210	p
Rubrobacter xylanophilus	600454	*Pseudomonas aeruginosa*	624114	p
Rubrobacter xylanophilus	600454	*Serratia marcescens*	622311	p
Rubrobacter xylanophilus	600454	*Streptococcus salivarius*	620690	p
Serratia marcescens	617614	*Agrobacterium tumefaciens*	617866	n
Serratia marcescens	623929	*Pseudomonas monteilii*	624642	n
Serratia marcescens	623929	*Streptococcus mitis*	624183	n
Serratia marcescens	617614	*Klebsiella pneumoniae*	623195	p
Serratia marcescens	622311	*Pseudomonas fluorescens*	624028	p
Serratia marcescens	622311	*Pseudomonas monteilii*	624642	p
Serratia marcescens	622311	*Sphingobium yanoikuyae*	623802	p
Sphingobium yanoikuyae	623802	*Staphylococcus cohnii*	623865	p
Staphylococcus cohnii	598217	*Acinetobacter nosocomialis*	606882	n
Staphylococcus cohnii	598217	*Acinetobacter radioresistens*	613572	n
Staphylococcus cohnii	598217	*Klebsiella oxytoca*	621896	n
Staphylococcus cohnii	598217	*Klebsiella pneumoniae*	620558	n
Staphylococcus cohnii	598217	*Klebsiella pneumoniae*	620675	n
Staphylococcus cohnii	598217	*Klebsiella pneumoniae*	623195	n
Staphylococcus cohnii	598217	*Pantoea dispersa*	619869	n
Staphylococcus cohnii	598217	*Pseudomonas fluorescens*	624028	n
Staphylococcus cohnii	623865	*Pseudomonas putida*	624672	n
Staphylococcus cohnii	598217	*Sphingobium yanoikuyae*	623802	n
Staphylococcus cohnii	598217	*Pseudomonas aeruginosa*	624114	p
Staphylococcus cohnii	598217	*Pseudomonas putida*	623836	p
Staphylococcus cohnii	598217	*Pseudomonas putida*	624672	p

Staphylococcus haemolyticus	591357	*Klebsiella pneumoniae*	623195	n
Staphylococcus haemolyticus	591357	*Methylobacterium radiotolerans*	623831	n
Staphylococcus haemolyticus	591357	*Pantoea dispersa*	599261	n
Staphylococcus haemolyticus	591357	*Porphyromonas bennonis*	584857	n
Staphylococcus haemolyticus	591357	*Pseudomonas aeruginosa*	624114	n
Staphylococcus haemolyticus	591357	*Pseudomonas oryzihabitans*	611399	n
Staphylococcus haemolyticus	591357	*Serratia marcescens*	622311	n
Staphylococcus haemolyticus	591357	*Serratia marcescens*	623929	n
Staphylococcus haemolyticus	591357	*Acinetobacter nosocomialis*	606882	p
Staphylococcus haemolyticus	591357	*Acinetobacter radioresistens*	613572	p
Staphylococcus haemolyticus	591357	*Agrobacterium tumefaciens*	617866	p
Staphylococcus haemolyticus	591357	*Bacillus cereus sp. group*	624484	p
Staphylococcus haemolyticus	591357	*Pseudomonas monteilii*	624642	p
Staphylococcus hominis	598572	*Pseudomonas plecoglossicida*	623690	n
Staphylococcus hominis	598572	*Pseudomonas aeruginosa*	624114	p
Staphylococcus warneri	621868	*Bacillus cereus sp. group*	624484	n
Staphylococcus warneri	621868	*Klebsiella oxytoca*	621896	n
Staphylococcus warneri	621868	*Methylobacterium radiotolerans*	623831	n
Staphylococcus warneri	621868	*Pseudomonas monteilii*	624642	n
Staphylococcus warneri	621868	*Pseudomonas stutzeri*	621954	n
Stenotrophomonas maltophilia	618830	*Escherichia coli*	624449	n
Stenotrophomonas maltophilia	618830	*Pseudomonas stutzeri*	621954	n
Streptococcus mitis	624183	*Bacillus cereus sp. group*	624484	n
Streptococcus parasanguinis	608270	*Klebsiella pneumoniae*	620558	n
Streptococcus parasanguinis	608270	*Pseudomonas stutzeri*	621004	n
Streptococcus parasanguinis	608270	*Escherichia coli*	624449	p
Streptococcus parasanguinis	608270	*Finegoldia magna*	618642	p
Streptococcus parasanguinis	608270	*Pseudomonas aeruginosa*	624114	p
Streptococcus salivarius	620690	*Bacillus megaterium*	624482	n
Streptococcus salivarius	620690	*Sphingobium yanoikuyae*	623802	n
Streptococcus salivarius	620690	*Staphylococcus warneri*	621868	n
Streptococcus salivarius	620690	*Pseudomonas plecoglossicida*	623690	p
